# Prefrontal influences on the function of the neural circuitry underlying anxious temperament in primates

**DOI:** 10.1093/oons/kvac016

**Published:** 2022-10-28

**Authors:** Margaux M. Kenwood, Jonathan A Oler, Do P M Tromp, Andrew S Fox, Marissa K Riedel, Patrick H Roseboom, Kevin G. Brunner, Nakul Aggarwal, Elisabeth A Murray, Ned H. Kalin

**Affiliations:** 1 Department of Psychiatry, Weill Cornell Medicine, New York, NY; 2 Psychiatry, Univ. of Wisconsin, Madison, WI; 3 Psychology, UC-Davis, Davis, CA; 4 Wisconsin National Primate Research Center, Univ. of Wisconsin, Madison, WI; 5 Section on the Neurobiology of Learning and Memory, Laboratory of Neuropsychology, NIMH, Bethesda, MD

**Keywords:** bed nucleus of the stria terminalis, DTI, FDG-PET, anxiety, nonhuman primate, orbitofrontal cortex

## Abstract

Anxious temperament, characterized by heightened behavioral and physiological reactivity to potential threat, is an early childhood risk factor for the later development of stress-related psychopathology. Using a well-validated nonhuman primate model, we tested the hypothesis that the prefrontal cortex (PFC) is critical in regulating the expression of primate anxiety-like behavior, as well as the function of subcortical components of the anxiety-related neural circuit. We performed aspiration lesions of a narrow ‘strip’ of the posterior orbitofrontal cortex (OFC) intended to disrupt both cortex and axons entering, exiting and coursing through the pOFC, particularly those of the uncinate fasciculus (UF), a white matter tract that courses adjacent to and through this region. The OFC is of particular interest as a potential regulatory region because of its extensive reciprocal connections with amygdala, other subcortical structures and other frontal lobe regions. We validated this lesion method by demonstrating marked lesion-induced decreases in the microstructural integrity of the UF, which contains most of the fibers that connect the ventral PFC with temporal lobe structures as well as with other frontal regions. While the lesions resulted in modest decreases in threat-related behavior, they substantially decreased metabolism in components of the circuit underlying threat processing. These findings provide evidence for the importance of structural connectivity between the PFC and key subcortical structures in regulating the functions of brain regions known to be involved in the adaptive and maladaptive expression of anxiety.

## INTRODUCTION

A behaviorally inhibited disposition, or anxious temperament (AT), is an early childhood temperament that is characterized by enhanced reactivity to potential threat and/or novelty. AT is stable across development and is linked to a 3- to 4-fold increase in the risk for the later development of anxiety disorders, depression and comorbid substance abuse [1–3]. To characterize the mechanisms underlying early-life pathological anxiety, we developed a nonhuman primate (NHP) model of AT. Our extensive studies in young NHPs using the no-eye-contact condition (NEC) of the human intruder paradigm in conjunction with fluorodeoxyglucose positron emission tomography (FDG-PET) imaging have characterized a distributed neural circuit in which variation in metabolism is associated with individual differences in AT [4–6]. The extended amygdala is at the core of the AT circuit [7, 8], and other components of the AT network include the anterior hippocampus, the medial thalamus and the periaqueductal gray, as well as prefrontal regions, such as the posterior orbitofrontal cortex (OFC), which is thought to provide regulatory input to the circuit’s subcortical components [9].

The OFC is of particular interest as a potential regulatory region because it communicates extensively with other frontal lobe sectors, integrating cognitive, sensory and affective information critical for the assessment of motivational salience and emotion regulation [10–14]. Decades of studying the effects of damage to the OFC, in humans and animal models, point to an important role for the OFC in the evaluation of potentially threatening stimuli [15–21]. In particular, work in NHPs demonstrates that aspiration lesions of the OFC affect motivational and emotional processes critical for adaptive behavioral responding [16–18, 22], and other studies in our laboratory also demonstrate that large OFC aspiration lesions reduce both AT and brain metabolism in the bed nucleus of the stria terminalis (BST) [9, 22].

While the effects of OFC aspiration lesions on AT and BST metabolism were previously attributed exclusively to the destruction of OFC neurons, it is possible that the behavioral and metabolic changes observed following these lesions reflect a combined effect of damage to neurons and to axonal fibers coursing through or near the OFC. These fibers provide structural connectivity among various prefrontal regions as well as between prefrontal regions and subcortical structures [23, 24] and other studies have drawn attention to the potential importance of these fibers in interpreting OFC lesion experiments [25]. Specifically, Rudebeck *et al*. [25] demonstrated different behavioral outcomes when the effects of OFC aspiration lesions were compared to the effects of neurotoxic lesions of the same target area, thereby implicating fibers passing through the lesion region. Ventral prefrontal white matter fibers, many of which form part of the uncinate fasciculus (UF), travel adjacent to and through the OFC [26–28], and it was damage to this fiber bundle that was postulated to underlie the alterations in emotion regulation reported in Rudebeck *et al*. [25]. Relevant to the translation of these findings, neuroimaging studies demonstrate alterations in the microstructural integrity of ventral prefrontal white matter, specifically in the UF, in patients with anxiety disorders [29–31] and with respect to AT in preadolescent male rhesus monkeys [32]. Together, these converging lines of evidence underscore the importance of the structural connectivity provided by these cortico-limbic fibers in conveying neural information relevant to the pathophysiological basis of stress-related psychopathology.

In the current study, we performed aspiration lesions of a narrow ‘strip’ of the posterior OFC in a sample of preadolescent females, a developmental stage in which AT and AT’s circuit are relatively stable across repeated testing [4] in both males and females. Indeed, AT emerges as a stable, temperamental marker after 3 months in rhesus monkeys, a time point that is roughly analogous to 1 year of age in humans [33]. By combining this lesioning strategy with multimodal neuroimaging, we sought to characterize the effects of this lesion on ventral prefrontal white matter tracts as well as on anxiety-related behavior and the function of subcortical structures underlying AT. A goal of this study was to explore the fiber-disrupting effects of the posterior OFC aspiration lesions and to understand the extent to which fiber disruption could explain previously observed behavioral and brain effects related to OFC lesions. Based on earlier work [9], we hypothesized that the strip lesions would be sufficient to reduce AT and other threat-related responses and that this would be accompanied by a decrease in threat-related metabolism in the BST and other components of the AT circuit.

## METHODS

### Subjects and surgical methods

An experimental timeline is presented in Supplementary Figure S1. Potential subjects were screened with a ten-minute exposure to NEC, and 20 female rhesus monkeys with mid-to-high levels of freezing were selected based on prior studies that demonstrated a decrease in defensive behaviors following OFC lesions [22, 25]. As can be seen in Supplementary Figure S2, 70% of the subjects selected fell into the upper quartile of a large sample (*n* = 721) of rhesus monkeys that underwent the 30-min NEC exposure as part of a variety of other studies performed in our laboratory. Of these 20 subjects (mean [± SD] age = 2.17 [± 0.29] years), half were randomly selected to receive bilateral posterior OFC strip lesions. The remaining animals served as age-matched, cage-mate controls. Animals were pair-housed under a 12-hour light/dark cycle at the Harlow Center for Biological Psychology following standard guidelines for animal care including daily enrichment. All procedures were performed according to the federal guidelines of animal care and use and with the approval of the University of Wisconsin-Madison Institutional Animal Care and Use Committee (IACUC). Surgical procedures were performed following previously published methods [25] and are described only briefly here. Animals were anesthetized with ketamine (up to 20 mg/kg, intramuscular, IM), administered atropine sulfate (up to 0.04 mg/kg, IM) to depress salivary secretion and prevent bradycardia, buprenorphine (0.01–0.03 mg/kg, IM, repeated every 6–12 hours) for analgesia and meloxicam (up to 0.2 mg/kg, subcutaneous, SC) for analgesia and its anti-inflammatory properties. Animals were intubated and received isoflurane anesthesia (1–3%, intratracheal) and were maintained on a ventilator. PlasmaLyte (up to 10 mg/kg/hr, intravenous, IV) was administered to maintain fluids and electrolytes. Cefazolin (20–25 mg/kg, IM or IV) was administered as a prophylactic antibiotic one day prior to surgery. Cefazolin was also administered immediately prior to surgery and then every 6 hours while under anesthesia. To reduce intracranial pressure and prevent brain swelling, mannitol (up to 2.0 g/kg, IV) was given. Vital signs including heart rate, respiration rate, oxygen saturation, end-tidal CO_2_ and body temperature were continuously monitored. Animals were placed in a stereotaxic frame and then prepared for surgery. Under sterile conditions and after incising and retracting cranial soft tissue, a large bone flap was raised to expose the bilateral frontal lobes. In a single procedure, one hemisphere at a time, an opening in the dura was created and the dural flap was reflected toward the orbit to allow access to the orbital surface of the brain. The brain was lifted with a blunt instrument to expose the ventral surface, and while being careful to avoid damaging the olfactory tract, a narrow ~3 mm strip of cortex was removed by a combination of electrocautery and aspiration using a surgical microscope. This narrow strip extended from the fundus of the lateral orbital sulcus through to the midline, at the caudal extent of the bilateral OFC (see Fig. 2a). Following confirmation of adequate lesions, the dura was closed, bone flap secured and the incision was closed in layers before animals were allowed to recover from anesthesia. Animals were given buprenorphine on the day following the surgery, meloxicam for up to 5 days following the surgery and an antibiotic for 5 days following the surgery to prevent infection. All drugs and treatments were given in consultation with veterinary staff.

### Behavioral phenotyping

#### AT –

Subjects were phenotyped for AT using their response to 30 min of the NEC condition of the Human Intruder Paradigm [34] both before and after the lesion. The subjects were placed in a standard testing cage (L: 76.5 cm × W: 71.5 cm × H: 79 cm) in a testing room. A human intruder entered and stood 2.5 m from the testing cage. The human intruder stood with their profile presented to the subject, avoiding direct eye contact and making all efforts to remain motionless. The 30 min of NEC were scored by an experienced observer using a closed-circuit audiovisual system. The observer was blind to group. Several behaviors were quantified, which have been described in detail in previous publications [5, 6, 35]. AT is a composite phenotype comprising two behavioral measures — freezing and cooing — as well as a physiological measure — blood cortisol. Freezing behavior is defined as the maintenance of a tense body posture for more than 3 s in the absence of other movements except slow head movements or eye blinks. ‘Cooing’ vocalizations are made by rounding and pursing the lips and have a characteristic frequency pattern, starting with an increase in frequency followed by a decrease. For collection of behaviorally relevant glucose uptake, animals were injected with FDG prior to administration of 30 min of NEC (see Brain Imaging Data Acquisition below). After completion of the 30 min of NEC, animals were anesthetized and blood was collected for cortisol assays, after which the animals were placed in the PET scanner. Plasma samples were assayed for cortisol using the MP Biomedicals (Solon, OH) Immuchem coated tube radioimmunoassay following the manufacturer’s instructions. Samples were assayed in duplicate and any samples that had coefficients of variation (CV%) > 20 were re-assayed. The intra-assay CV% was 4.9 and the inter-assay CV% was 10.3. The detection limit defined by the lowest standard was 1 *μ*g/dl. To mitigate any potential effects of time of day on cortisol, all testing was done between 8:00 AM and 11:30 AM.

#### Novel Conspecific Test –

Testing for the novel conspecific paradigm occurred on a single day, with no pre-training. An older, larger, more dominant female, referred to as the ‘stimulus animal,’ was placed in a testing cage (L: 153 cm × W: 71.5 cm × H: 79 cm). The stimulus animal was allowed to habituate to the cage for 5 min, after which a metal dividing panel was inserted to separate the testing cage into two compartments of equal size, one of which contained the stimulus animal. The test animal was introduced into the other compartment and allowed to habituate for ~2 min prior to removal of the dividing panel. After the divider was removed, the animals were allowed to freely interact for 30 min. Dyadic behavior was scored by a rater blind to condition using a rating scale focusing on agonistic and affiliative behaviors [36]. Nonsocial behaviors, such as freezing and locomotion, and vocalizations, such as cooing and barking, were also scored.

#### Snake Fear Test –

On the first day of training, referred to as cage adaptation, subjects were placed in the Wisconsin General Testing Apparatus (WGTA) environment for 60 min. A clear plastic stimulus box (56 × 21 × 6 cm) with different food rewards was placed within the animal’s reach during the cage adaptation period. Food rewards were restocked at 15-min intervals during adaptation only if the animal had cleared all treats offered on the box. On the second and third day of training, food preferences for each subject were determined by recording the order in which subjects selected food rewards. Trials during this phase ended either when the subject had retrieved all food rewards or 60 min had elapsed. On the 4th day, referred to as reach adaptation, subjects were trained to reach for their preferred food rewards. Based on the order determined, the two most preferred rewards were placed either on the left or right distant corner of the testing box. In order to proceed to the testing phase, subjects had to retrieve at least one food reward in a minimum of 20 out of 24 trials. If the subject failed to reach in 20 out of 24 trials, this stage of training was repeated until subjects reached criterion. During snake fear testing, animals were presented with their two most preferred food rewards on the two distant corners of the stimulus box (left and right). This required the animals to reach directly over the box and over any stimulus present inside the box to retrieve a food reward. During testing, one of four stimuli was presented inside the box: (i) nothing: the empty box; (ii) tape: roll of blue masking tape; (iii) rubber snake: curled green rubber snake, 110-cm long; and (iv) snake: live California king snake (*Lampropeltis getula californiae*), 132-cm long. Each stimulus was presented six times throughout the course of testing in a pseudorandom order with the following stipulations: (i) The real snake was not presented during the first five trials and (ii) no stimulus was presented for more than two consecutive trials. Testing order was the same across all monkeys. Trials lasted for 60 s (regardless of subject response) with an inter-trial interval of 45 s. All WGTA training and testing took place between 10:30 AM-04:30 PM. The latency of the animal’s first reward retrieval in each trial was used for analysis.

### Statistical analysis of behavioral data

#### NEC behavior:

To be consistent with prior studies [5, 6], several transformations were applied to normalize the behavioral data. Mean freezing duration was log transformed and cooing frequency was square root transformed. Standardized cortisol, freezing and vocalization measures were created by z-scoring variables across pre- and post-time points. Normality tests on the transformed, normalized variables were performed. Because so few subjects displayed cooing behavior, violating the normality assumption (Shapiro–Wilk test, W = 0.4; *P* < 0.0001), we excluded cooing from our AT calculation. Because of the small sample size and narrow age range, we elected not to control for age in the AT calculation. Similarly, because of the narrow time range for cortisol collection (between 9:00 AM and 11:00 AM for all subjects), we did not correct threat-related cortisol measurements for time of day. Transformed freezing and cortisol were averaged to determine each subject’s AT score. A repeated-measures ANOVA (group × time; controlling for age) was used to examine lesion-related changes AT.

#### Novel conspecific test:

To avoid confounds related to habitation, the novel conspecific test was only performed once, during the postsurgical testing. A *t*-test was used to examine between-groups differences in freezing behavior during exposure to the stimulus animal.

#### Snake fear test:

To avoid confounds related to habitation, adaptation to the testing environment, training to reach and the snake fear test were only performed during the postsurgical testing. Differences in the time animals took to habituate to the testing environment were examined by comparing the average slope of the reach latencies across the 24 trials of the first reach adaptation training day with a one-way ANOVA while controlling for age. A repeated-measures ANOVA (trial × object × group) was used to examine lesion-related differences in the response to the stimuli used in the snake fear test. All analyses were performed using the pingouin package (https://pingouin-stats.org/, [37]) in Python 3.6 and in IBM SPSS Statistics (version 27).

### Brain imaging data acquisition

All study subjects were scanned with MRI (including T1-weighted and diffusion-weighted images) and FDG-PET both before and after surgery (experimental group) or rest (unoperated controls). Animals were first assessed using FDG-PET imaging an average of 6.8 (±0.46) weeks prior to surgery. MRI data were collected roughly a week after the PET scan, averaging 5.5 (±0.71) weeks prior to surgery. Post-surgical FDG-PET scans were collected an average of 16.23 (±1.5) weeks after surgery, allowing sufficient time for recovery. Post-surgical MRI scans were collected an average of 17.37 (±1.46) weeks after the surgery. For one animal, technical difficulties led to the loss of behavioral NEC data collected as part of the postsurgical FDG-PET test. FDG-PET scanning in combination with NEC was performed again for this subject and its cage mate control, and those data were used for behavioral analyses.

PET scanning requires general anesthesia, and animals were food fasted the night before FDG administration and testing in the NEC. To measure threat-related metabolic activity animals were injected intravenously with FDG (~7.0 mCi) and placed in a test cage for 30 min, during which time the animals were exposed to NEC. After the 30-min FDG-uptake period, animals were anesthetized with 15-mg/kg ketamine and 0.04-mg/kg atropine and fitted with an endotracheal tube to maintain isofluorane (1–2%) anesthesia throughout the scanning procedure. Vital signs, including heart rate, oxygen saturation, end tidal CO_2_, respiration rate and body temperature, were monitored throughout the scan. Data were collected using a Focus 220 microPET scanner (Siemens Microsystems, Knoxville, TN). Sixty-minute emission PET scans were reconstructed using standard filtered back projection methods and reflect the integrated brain metabolism that occurred during the 30 min of FDG uptake.

Prior to MRI acquisition, animals were anesthetized with ketamine (15 mg/kg, IM), placed in an MRI-compatible stereotaxic frame, administered with dexmedetomidine (15 ug/kg, IM) and scanned for ~1 hour. Heart rate and oxygen saturation were monitored throughout the course of the scan. Additional doses of ketamine were given as needed throughout the scan to maintain anesthesia. At the conclusion of the scan, atipamezole (0.15 mg/kg) was administered to reverse the effects of dexmedetomidine and animals were removed from the scanner and monitored until fully recovered from anesthesia. Scans were collected using a 3-Tesla GE 750 (GE Healthcare; Waukesha, WI, USA) MRI scanner with a custom 8-channel array coil (Clinical MR Solutions; Brookfield, WI, USA).

Whole-brain anatomical images were acquired using an coronal T1-weighted 3D inversion recovery fast spoiled gradient recalled scan [IR-fSPGR; repetition time (TR) = 11.9 ms, echo time (TE) = 5.4 ms, inversion time (TI) = 600 ms, flip angle = 10°, number of excitations (NEX) = 2, field of view (FOV) = 140 mm, matrix = 256 × 224 interpolated to 512 × 512, in-plane resolution = 0.27 mm, slice thickness/gap = 0.5/0 mm, 248 slices].

Diffusion weighted images (DWI) were collected using a 24 echo-planar, spin-echo sequence (TR/TE = 10 000/85.3 ms, flip angle = 90°, NEX = 1, FOV = 144 mm, matrix = 128 × 128 interpolated to 256 × 256, in-plane resolution = 0.5625 mm, slice thickness/gap = 1.3/0 mm, 68 interleaved slices, echo-planar spacing = 816 *μ*s. DWI at b = 1000 s/mm^2^ was performed in 72 non-collinear directions with 6 non-diffusion-weighted images). Images were acquired in the coronal plane through the entire monkey brain. A co-planar field map was also obtained for the diffusion-weighted images, using a gradient echo with images at two echo times: TE1 = 7 ms, TE2 = 10 ms.

### Coregistration and pre-processing of imaging data

We corrected for low frequency intensity non-uniformity that often presents in MRI imaging by applying a bias field correction using N3BiasFieldCorrection. The longitudinal normalization pipeline was adapted to accommodate the lesioned brain regions and associated brain shifts. As such, all pre-lesion T1 images were iteratively normalized using affine and nonlinear warps to a previously published 592-monkey template [5] using Advanced Normalization Tools (ANTs, [38, 39]). Then, we ran a rigid body registration of the post-lesion T1 images to the pre-lesion T1 images in native space using FLIRT with 6 degrees of freedom [40]. Finally, to warp the post-lesion images to template space, the pre-lesion affine and warp files were applied to the post-lesion T1 images in pre-lesion space.

Gray matter probability (GMP) was estimated and extracted in template space using FMRIB’s Automated Segmentation Tool [41]. The pre-lesion GMP map was subtracted from the post-lesion GMP and spatially smoothed with a 4-mm FWHM Gaussian kernel.

Each native-space FDG-PET image was linearly aligned to its corresponding T1 using a rigid-body transformation in ANTs and then aligned to the 592-monkey template space using the T1 → template transformation described in the paragraph above. The normalized FDG-PET images were scaled to the whole-brain signal using FSL [42]. The grand-mean scaled Post-lesion PET images were subtracted from Pre-lesion scaled PET images and then all scaled pre, post, [post - pre] PET images were spatially smoothed with a 4-mm FWHM Gaussian kernel.

DWI scans were processed as previously described in [32]. Briefly, images were corrected for field inhomogeneities and eddy currents, after which the tensors were calculated using a robust tensor estimation in Camino [40]. Pre-surgery diffusion tensor images of all subjects were coregistered iteratively using nonlinear tensor-based normalization tools (DTI-TK; [41]), and then registered to the 592-monkey T1-template. Pre- and post-surgery scans were coregistered within each subject, such that the warp from presurgical native-space to 592 T1-template could be applied to the post-surgery scans as well. This was done to ensure that the final template would not be distorted by the posterior OFC strip lesions. In this template space fractional anisotropy (FA) was extracted to quantify local white matter microstructure. [Post – pre] images were calculated and smoothed with a 4-mm FWHM Gaussian kernel.

A DTI population average was created from pre-surgery and post-surgery images separately. Whole-brain fiber tractography was performed using Camino, using a tensor deflection algorithm. Fiber tracking was terminated in voxels where FA was below 0.1, or where the curvature angle between consecutive streamline steps was more than 90 degrees. The visualization software TrackVis (trackvis.org) was used to delineate the primary white matter tract of interest, the UF, using anatomically defined waypoints [43–45]. More specifically, the most posterior coronal section that showed clear separation of the frontal and temporal lobes bilaterally was identified in the population average pre-surgery FA image. Bilateral frontal and temporal lobe seed regions of interest were then manually drawn on this coronal section. The Boolean AND term was used to select only fibers that crossed through both the temporal and frontal seed regions of interest.

### Lesion characterization with T1 images

In order to get an impression of the extent of the strip lesion both DPMT and NA used the post-surgery T1 scans to manually identify and draw the areas of the brain that were aspirated during the surgery. The lesion masks for each subject were warped to template space and added together to create a population lesion mask. It should be noted however that identification of the lesion size and extent was complicated by the brain shifting into the aspirated region.

### Statistical analysis of imaging data

Voxel-based morphometry (VBM) analysis: T1-weighted structural MRI data were used to examine posterior OFC strip lesion-induced reductions in brain volume, controlling for age, with a voxel-wise regression analysis. A 3D statistical map of the [post - pre] change in GMP was created, and regions demonstrating significant group differences (lesion_[post-pre]_ – control_[post-pre]_) were identified. Multiple comparison correction was applied using a threshold-free cluster enhancement (TFCE) [46] to identify statistically significant alterations in brain volume.

Diffusion tensor imaging (DTI) analysis: To examine posterior OFC strip lesion-induced changes in white matter microstructure, we performed whole-brain fiber tractography and used anatomical way points to identify fiber tracts of interest. To examine alterations in UF integrity, average FA was extracted from the bilateral UF region of interest (ROI), defined by the tractography from both pre- and post-lesion scans, and a repeated measures ANOVA (group × time), controlling for age, was used to test for group differences in UF FA. We examined the correlation between [post - pre] changes in UF FA and changes in AT using Pearson’s correlation coefficient (r).

In addition to the tract-based analyses, we also conducted a two-tailed voxelwise regression, controlling for nuisance variance associated with age, to examine posterior OFC strip lesion-induced changes in whole brain FA. Three-dimensional brain maps of the [post - pre] change in FA were created, and regions demonstrating significant group differences (lesion_[post-pre]_ – control_[post-pre]_) were identified using the TFCE method. The resulting statistical brain map was corrected using family-wise error (FWE) correction (*P* < 0.05).

Brain metabolism (FDG-PET) analysis: To test our a priori hypothesis regarding the effects of posterior OFC strip lesions on BST function, we extracted the mean FDG signal from an anatomically defined BST ROI. This ROI was drawn based on the anatomical boundaries defined in a rhesus brain atlas [47]. The mean signal was extracted from all voxels within the BST ROI and a repeated-measures ANOVA (group × time), controlling for age, was used to assess lesion-induced changes in BST metabolism. This analysis was repeated in the amygdala, with an ROI drawn based on the anatomical boundaries defined in the rhesus brain atlas [47].

In addition to the ROI-based analyses, we also conducted a two-tailed voxel-wise regression analysis that controlled for the change in GMP at each voxel and age. Three-dimensional brain maps of the [post - pre] change in the scaled PET signal were created, and regions demonstrating significant group differences (lesion_[post-pre]_ – control_[post-pre]_) were identified TFCE. The resulting statistical brain map was corrected using FWE correction (*P* < 0.05).

## RESULTS

Subjects were assessed in a variety of paradigms focused on fear- and anxiety-related behaviors. While the posterior OFC strip lesions resulted in modest decreases in threat-related responding, the lesions produced marked effects on brain metabolism in components of the circuit underlying AT, particularly in the BST. These metabolic alterations were accompanied by lesion-induced white matter alterations in the UF, a white matter tract that connects prefrontal and temporal lobe structures.

### OFC lesion-induced behavioral effects

#### No-eye-contact condition

**Figure 1.**
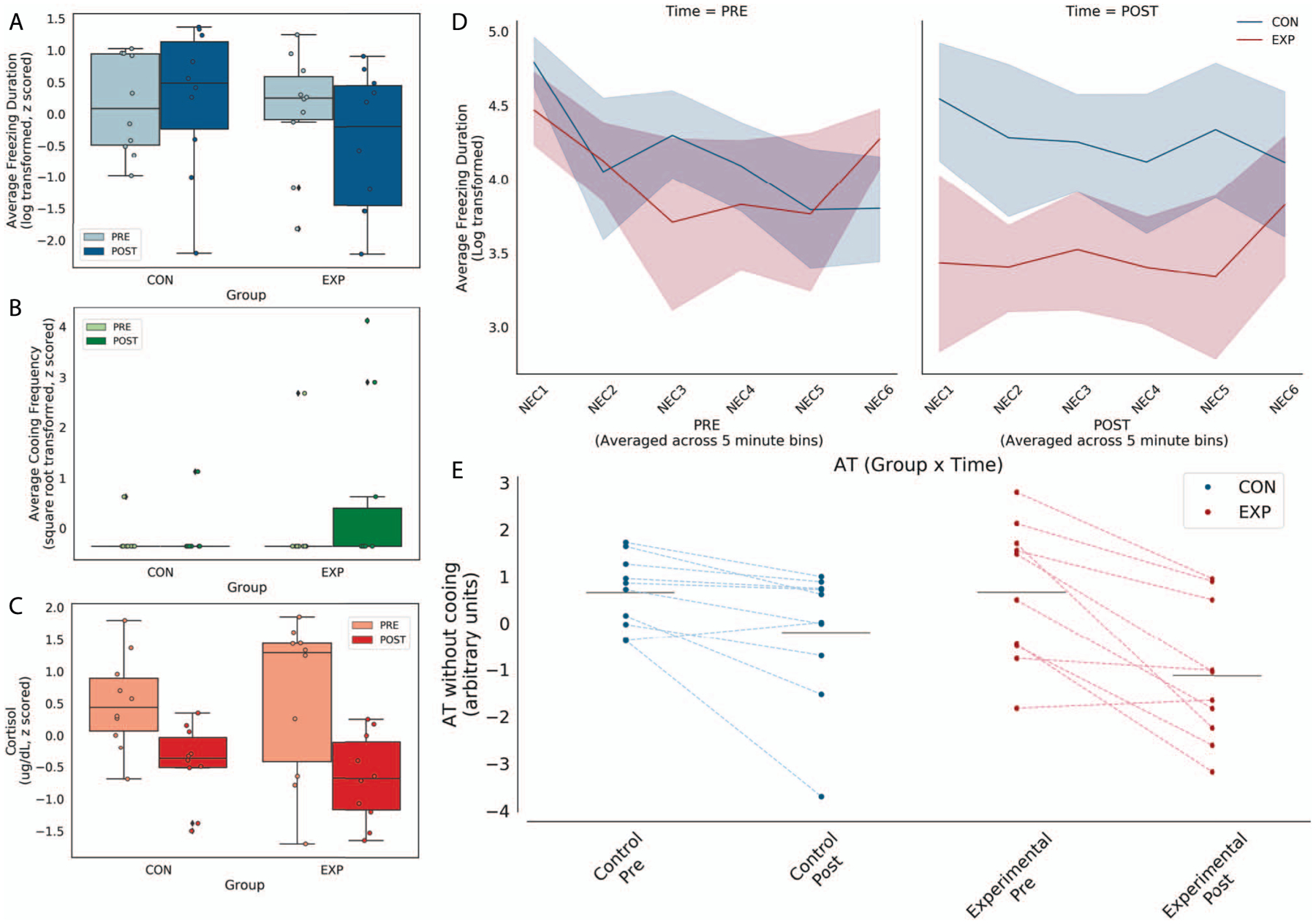
Posterior OFC strip lesions decrease AT-related behavioral responses during NEC. Left panel: NEC-related in freezing (top), cooing (middle) and cortisol (bottom). Because few animals demonstrated any cooing, this variable was excluded from the AT score. Right panel: Freezing across 5-min bins within the NEC is shown in the top panel at the pre- and post-lesion time points. Of note, the lesioned animals froze less than controls across all 5-minute bins. In the bottom panel, AT calculated without cooing from the pre and post time points, with lines connecting each subject’s pre and post AT score.

AT was assessed prior to and after the surgery to examine lesion-induced changes in AT and, based on our previous work with large OFC aspiration lesions, we anticipated a lesion-induced decrease in AT [9, 22]. As very few of the subjects displayed cooing behavior (Fig. 1b), we calculated AT as the average of NEC-induced freezing and cortisol, omitting cooing. A repeated-measures ANOVA (group × time), controlling for age, demonstrated a group × time interaction that trended toward but did not reach statistical significance (*F*(1,17) = 2.93, *P* = 0.11, partial η^2^ = 0.15, Fig. 1e), such that the lesioned animals demonstrated a more substantial decrease in AT relative to controls. The direction of this effect is consistent with previous OFC lesion studies reporting a decrease in threat-related responses following large aspiration lesions of the OFC [22].

We next examined the effects of the posterior OFC strip lesions separately on NEC-related freezing and cortisol, the two components that were used to calculate AT in this study. A repeated-measures ANOVA (group × time), controlling for age, demonstrated that the majority of the subjects showed a decrease in freezing between the first (pre) and second (post) exposure to the NEC (main effect of time: *F*_(1,17)_ = 8.00, *P* < 0.01, partial η^2^ = 0.32), suggestive of habituation across the testing sessions. This analysis also revealed that there was not a significant effect of the lesions on freezing (group × time: *F*_(1,17)_ = 2.06, *P* = 0.17, partial η^2^ = 0.11, Fig. 1a) between the pre- and post-lesion time points. To assess whether the posterior OFC-lesioned subjects displayed differences in habituation across the 30-min exposure following the lesion, freezing duration at the post lesion test was binned in 5-min increments. The posterior OFC-lesioned animals displayed less freezing during each 5-min bins of the 30-min NEC exposure (Fig. 1d). Post-hoc *t*-tests revealed the difference between the controls and experimental animals was statistically significant only for bin 2 (*t*_(9)_ = 3.06, *P* = 0.014).

With respect to threat-related cortisol, a repeated-measures ANOVA (group × time), controlling for age, demonstrated a main effect of time that trended toward but did not reach statistical significance (main effect of time: *F*_(1,17)_ = 4.10, *P* < 0.06, partial η^2^ = 0.19) such that the majority of the subjects showed a decrease in plasma cortisol levels between the first (pre) and second (post) exposure to the NEC, suggesting habituation across the testing sessions. This analysis also revealed that there was not a significant effect of the lesions on cortisol levels (group × time: *F*_(1,17)_ = 0.15, *P* = 0.71, partial η^2^ = 0.01, Fig. 1c) between the pre- and post-lesion time points.

#### Novel conspecific paradigm

We next examined the effects of the posterior OFC strip lesions on behavior in the ‘novel conspecific’ paradigm, which allows for an assessment of fear and anxiety elicited during a threatening social interaction. Compared to controls, the monkeys with the posterior OFC strip lesions engaged in less freezing behavior when confronted by the novel, older animal, an effect that trended toward significance (*t*_(17)_ = 3.76, *P* = 0.068). Thus, consistent with the modest reduction in AT, monkeys with posterior OFC strip lesions showed decreased defensive responses when confronted by a novel conspecific.

#### Snake fear

Rhesus monkeys are naturally fearful of snakes [48], and both the control and lesion animals displayed significantly increased latencies to reach for a food reward in the snake fear test: presence of a real snake > fake snake > a neutral stimulus > no stimulus (main effect of object, *F*_(3,57)_ = 27.93, *P* < 0.0001, partial η^2^ = 0.59; see Supplementary Fig. S3). Based on results from our earlier large OFC aspiration lesion study [22] and those from a smaller group of monkeys with strip lesions similar to those in the present study [25], we anticipated a lesion-related difference in the response to the snake. However, in contrast to our expectations, the monkeys with posterior OFC strip lesions did not significantly differ from controls in their latencies to reach for a food reward in the presence of the snake (object × group interaction, *F*_(3,54)_ = 0.941, *P* = 0.427, partial η^2^ = 0.05).

Due to methodological differences in testing between this and previous studies [18, 25], we are cautious in interpreting these null results. One study reported that the effects of OFC aspiration lesions on the snake fear test are more pronounced on later days of testing, relative to the initial exposure [49]. If so, perhaps in our study continued testing would have revealed a group difference. Consistent with this idea, although no group differences were observed in the snake-fear test, on the first day of adaptation for this test (monkeys were trained to reach for food rewards in the absence of a specific threat; see Methods), the control monkeys habituated to the unfamiliar conditions of the testing environment, showing progressively shorter retrieval latencies to reach for a food reward, whereas the lesioned monkeys did not demonstrate habituation, appearing to have a steady initial response pattern (mean habituation slope, control vs. lesion, controlling for age, *F*_(1,17)_ = 7.696, *P* = 0.013, partial η^2^ = 0.31; see Supplementary Fig. S4).

#### Characterization of the OFC strip lesions

**Figure 2.**
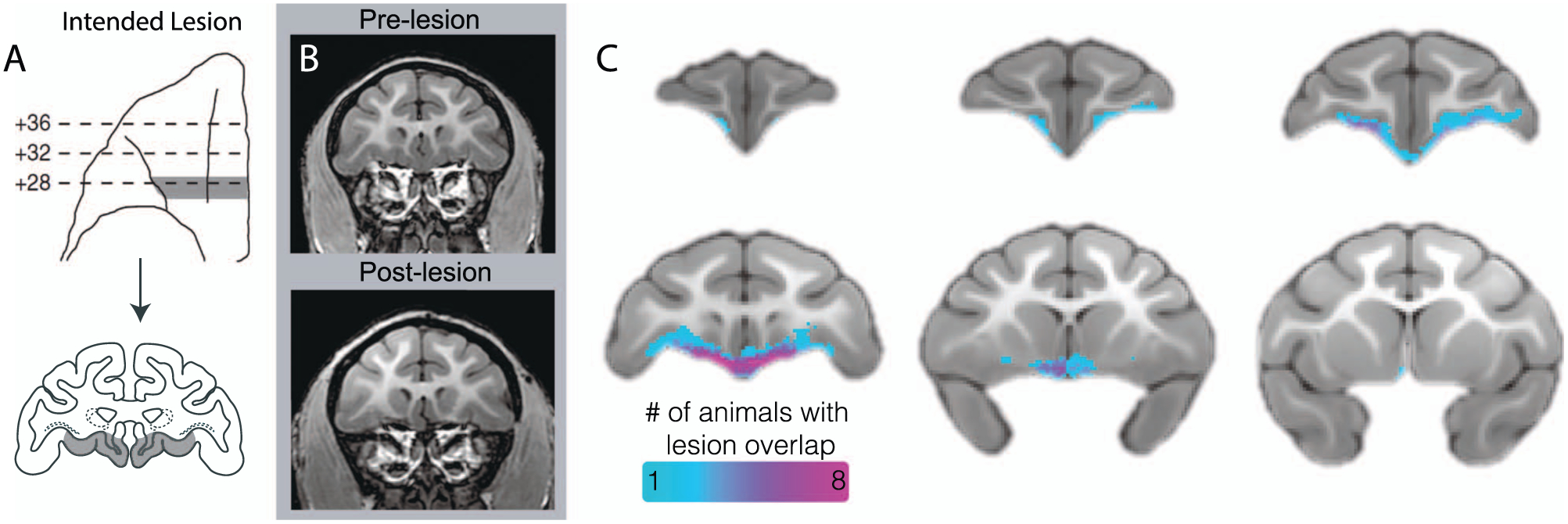
Assessment of posterior OFC strip lesion extent with structural neuroimaging. (a) Bilateral lesions targeted an 3-mm strip of cortex toward the caudal extent of the OFC, extending from the lateral orbital sulcus to the midline. Drawings indicate the intended lesion in gray (images reproduced from Rubebeck *et al*., 2013, with minor modifications). (b) The pre- and post-surgical T1 images demonstrate, in a representative animal, tissue loss in the lesion region. (c) An aggregate map of the manual tracing of each experimental subject’s lesion based on T1 images demonstrates an *in vivo* characterization of lesion extent.

As can be seen in Fig. 2a, the lesions targeted a narrow, 3-mm strip of OFC that extended from the fundus of the lateral orbital sulcus to the rostral sulcus on the midline, including the caudal extent of Walker’s area 13 and portions of areas 14 and 25. T1-weighted images comparing pre-lesion to post-lesion scans indicated tissue loss in the targeted region, affecting a strip of cortical gray matter as well as some of the adjacent dorsal and medial white matter (see Fig. 2b). To examine individual differences in the size and extent of the lesion, manual tracing was performed on the post-lesion T1 scans. The aggregate map can be seen in Fig. 2c, showing that the lesions primarily targeted a narrow strip of cortex, encompassing the posterior portions of area 13 and portions of adjacent area 25. In an exploratory analysis, we evaluated the relationship between the volume of the lesion and both the pre-post difference in AT and AT measures obtained after surgery, to determine if the size of the lesion was associated with the behavioral change. Interestingly, neither the change in AT (*R*^2^ = 0.009, *P* = 0.79) nor the AT scores at the post time point *R*^2^ = 0.006, *P* = 0.82) were correlated with the manually estimated lesion size.

To further characterize volumetric changes linked to the lesion, we assessed whole brain GMP and there were highly significant reductions in tissue volume in the targeted posterior OFC region. Other cortical (dorsolateral and ventrolateral prefrontal cortex [PFC]) and striatal (caudate and putamen) regions also showed volume reductions (see Supplementary Table S1). To control for the effects of either tissue shifts and/or gray matter degeneration in relation to analyses of glucose metabolism, the GMP at each voxel was controlled for in all analyses [50].

#### Assessing fiber disruption resulting from the OFC strip lesions

**Figure 3.**
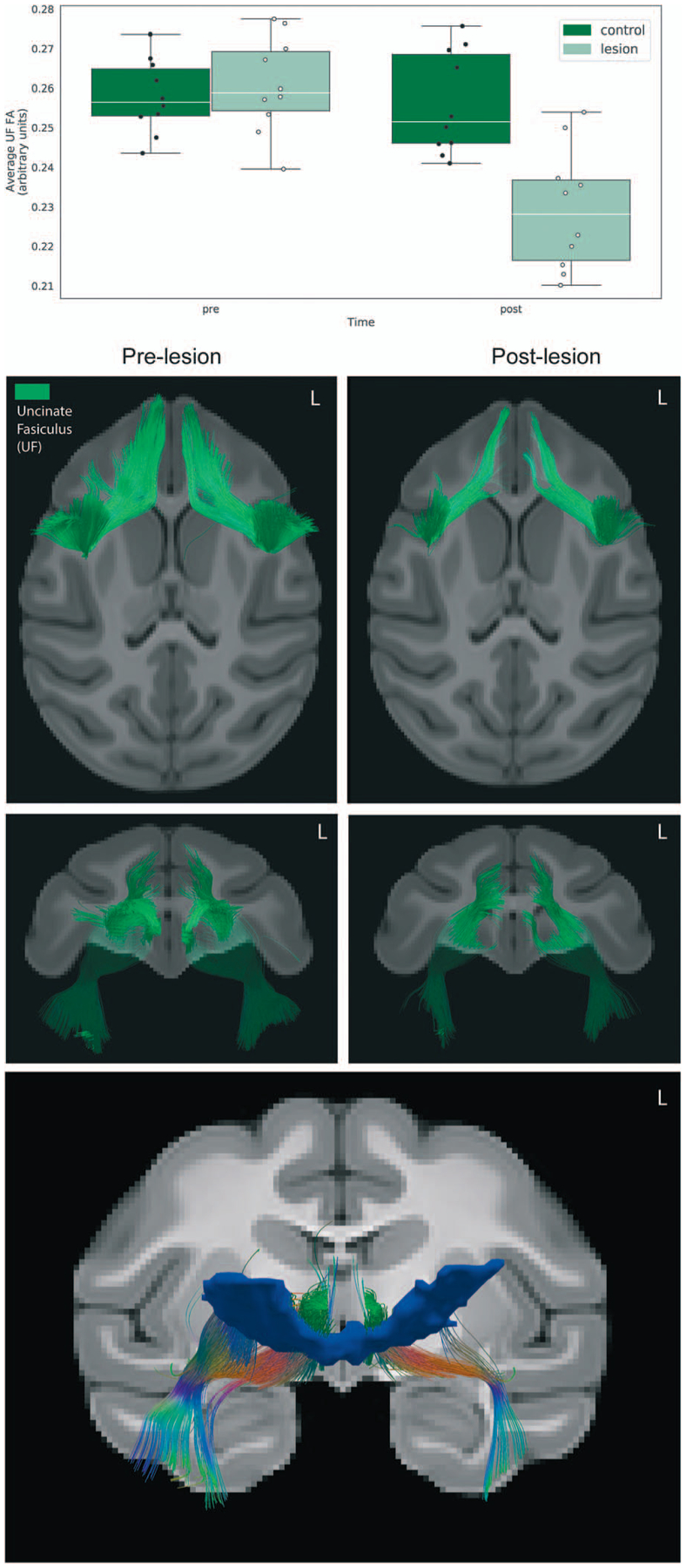
Effects of the lesions on uncinate fasciculus (UF) microstructure. (Top) Bar graph depicting the average FA change across the entire UF ROI. (Middle) Deterministic tractography of the UF comparing the pre-lesion to the post-lesion tractography in the 10 experimental animals confirms the white matter damage, as can be seen in the axial and coronal views. (Bottom) Using the lesion mask shown in Fig. 2c as a waypoint ROI, fibers traveling through the lesion region at the pre time point were delineated using deterministic tractography.

We used tensor-based deterministic tractography to qualitatively characterize the effects of the lesions on the white matter tracts passing nearby and/or through the posterior OFC, specifically the UF. As can be seen in Fig. 3, tractography revealed bilateral disruptions of fibers in the medial aspects of the frontal portions of the UF. To quantify effects on the UF, we extracted mean FA values from the bilateral UF ROI and performed a repeated measures ANOVA (group × time; covaried for age), which demonstrated a significant interaction, such that the monkeys with posterior OFC strip lesions had marked reductions in UF FA relative to unoperated controls (*F*_(1,17)_ = 28.08, *P* < 0.0001, partial η^2^ = 0.61). Additionally, when controlling for the effect of group, there was a significant association between the average FA change within the UF and the change in AT (Pearson’s *r* = 0.46, *P* = 0.04).

In an exploratory analysis, the population lesion mask generated from the manual tracing of the lesion in each subject was used as a way point, to explore tracts that traveled through the lesioned region. As can be seen in Fig. 3, fibers that overlap with regions of the UF and inferior fronto-occipital fasciculus are crossing through the lesioned region. Although sparse, we also observe fibers that roughly overlap with the accumbofrontal fasciculus (AFF). The AFF, which has been shown in humans to connect the ventromedial PFC with striatal regions [51], may also carry fibers connecting the ventral PFC with the BST.

#### Effects of posterior OFC strip lesions on whole-brain white matter microstructure

ROI-based analyses can be limited as they average signal changes across large regions, potentially masking subregional differences within fiber tracts or across non-delineated portions of white matter. To explore lesion-induced microstructural changes throughout the brain, a voxel-wise analysis was performed [lesion_[post-pre]_ – control_[post-pre]_] on the DTI data (see Fig. 5, bottom panel). In addition to confirming decreased FA within regions of the UF tract, this analysis also revealed several additional clusters of voxels with reduced FA. Notably, within the largest of these clusters, the anterior limbs of the internal capsule (IC) demonstrated the most highly significant lesion-induced changes in FA (see Supplementary Table S2). This portion of the IC carries fibers traveling from the ventral PFC to the brainstem and thalamus [24, 52], as well as fibers posited to travel toward the BST [53]. We also observed several regions with significant post-lesion increases in FA, mostly restricted to gray matter, particularly of the dorsolateral PFC (see Fig. 5, bottom panel, and Supplementary Table S2). While it seems likely these changes were caused by the lesions, we are hesitant to interpret these unexpected results as we cannot disentangle whether these changes reflect compensatory mechanisms within distal white matter pathways, or possibly gray matter degradation in the cortex resulting from lesion-induced deafferentation.

#### Effects of OFC strip lesions on BST metabolism

**Figure 4.**
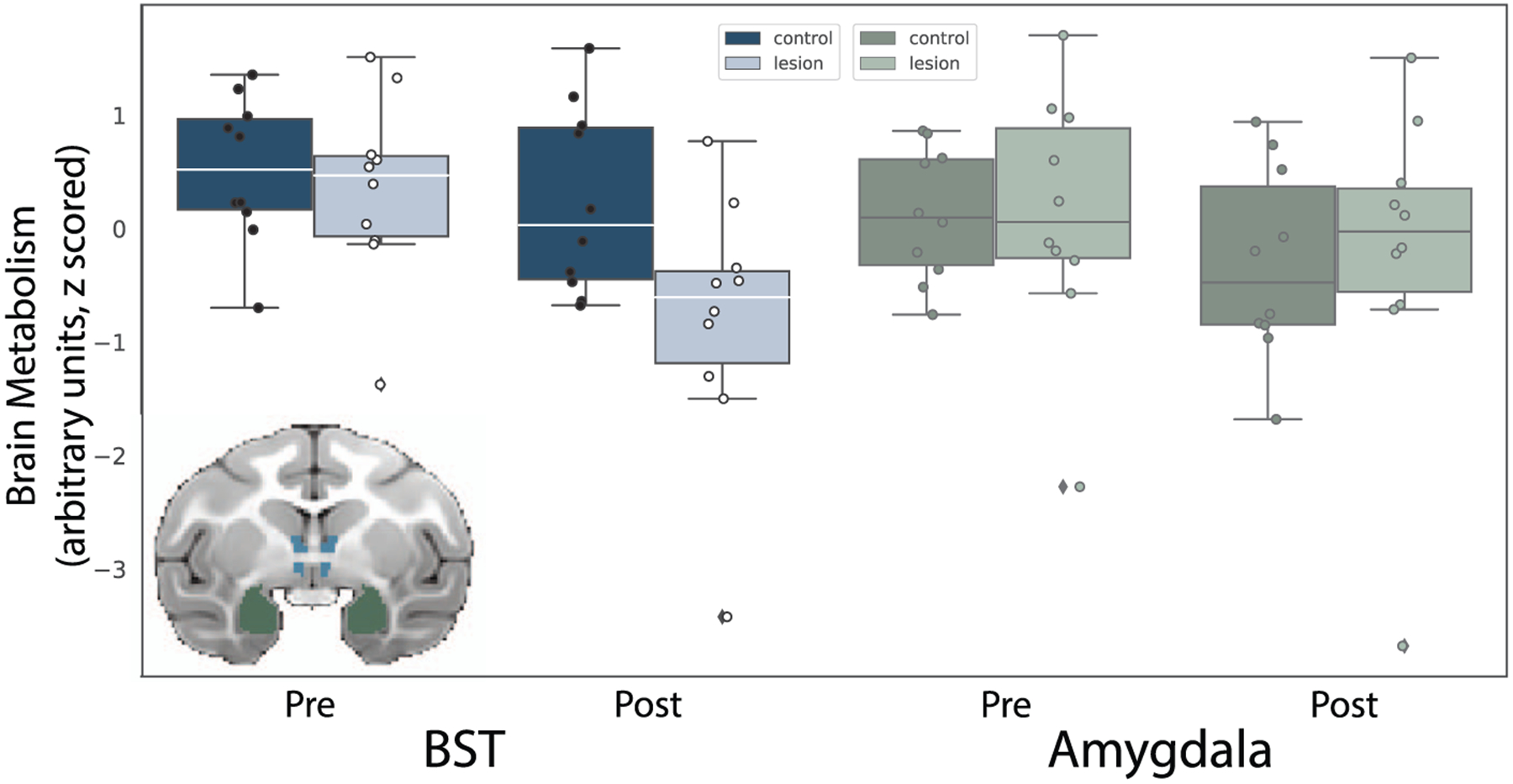
Lesion-induced changes in BST and amygdala metabolism. The inset shows the ROIs used to extract average BST (blue) and amygdala (green) brain metabolic activity, to compare the pre-lesion to post-lesion FDG-PET uptake (controlling for GMP) in monkeys with posterior OFC strip lesions relative to unoperated controls. The bar plots show data points denoting each subject’s BST and amygdala glucose metabolism at both time points (presented with arbitrary units, scaled to the whole brain).

Based on our previous findings with large aspiration lesions of the OFC [9], we hypothesized that damage to the posterior OFC, including neurons and fibers passing nearby and/or through the posterior OFC, would result in altered BST function. A BST ROI (see Fig. 4, inset) was used to compare the pre-lesion to post-lesion FDG-PET uptake in monkeys with posterior OFC strip lesions relative to unoperated controls (Fig. 4, left). Mean metabolism values for this ROI were extracted and corrected for age and mean GMP within the ROI. A repeated-measures ANOVA (group × time), controlling for age, revealed a group × time interaction (*F*_(1,17)_ = 4.49, *P* = 0.05, partial η^2^ = 0.21). Post-hoc testing revealed that BST metabolism was significantly decreased following the posterior OFC strip lesion (*t*_(9)_ = −5.84, *P* < 0.001). This difference was not observed in the control subjects (*t*_(9)_ = −0.311, *P* = 0.76). Metabolism data from an anatomically defined amygdala ROI were also extracted (see Fig. 4, inset) and subjected to the same analysis. The posterior OFC strip lesions did not appear to influence amygdala metabolism (see Fig. 4, right). The comparative effect on BST and amygdala metabolism was tested using a repeated-measures ANOVA (group × time × region), which revealed a significant group × time × region interaction (*F*_(1,17)_ = 17.00, *P* = 0.002, η^2^ = 0.43).

#### Effects of OFC strip lesions on whole-brain glucose metabolism

**Figure 5.**
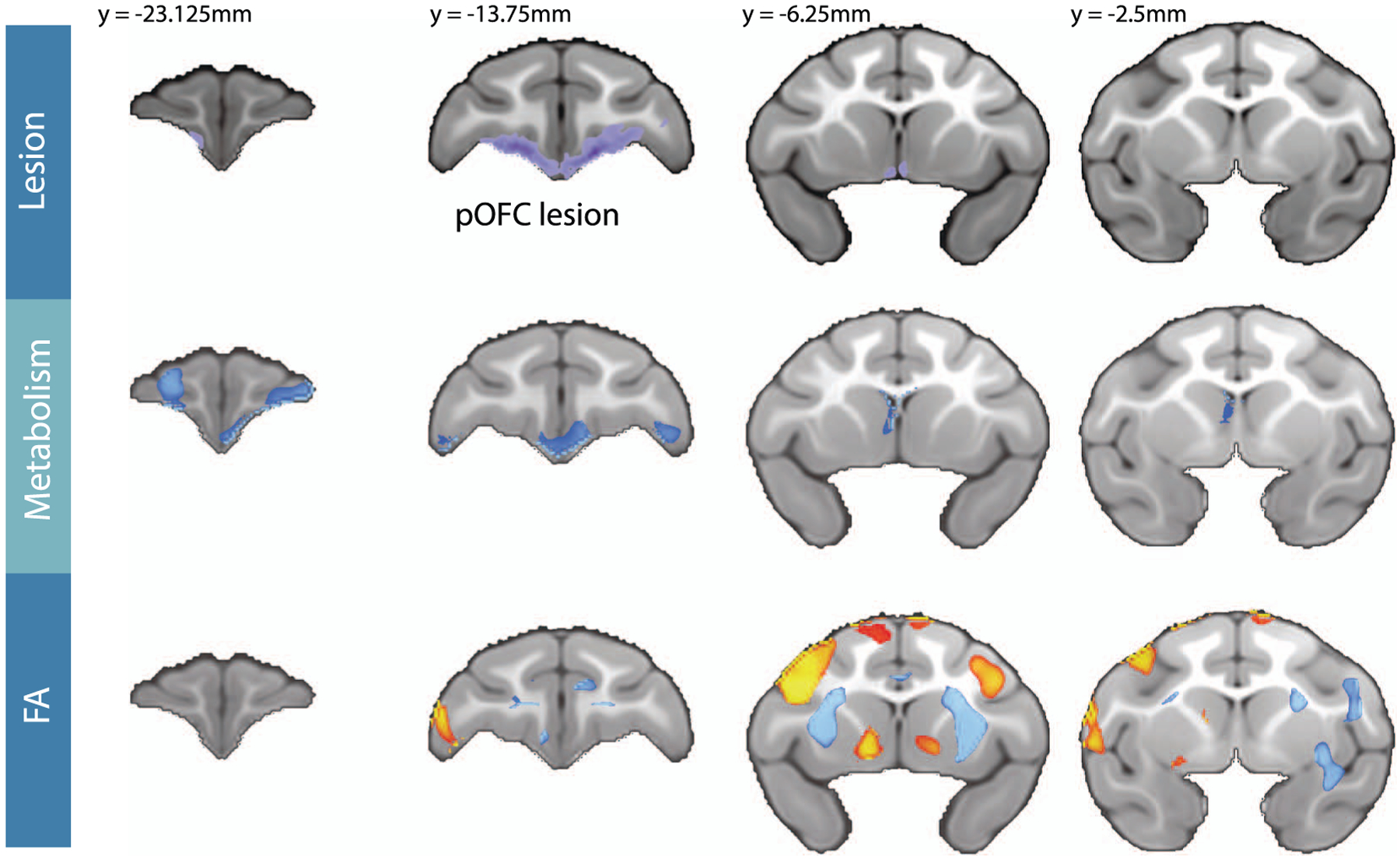
Changes in FDG metabolism and FA resulting from the posterior OFC lesion. The top panel shows the average lesion extent across all lesioned subjects, which was manually traced using T1 weighted images. The remaining two panels show the [lesion_[post-pre]_ – Control_[post-pre]_] changes in FDG metabolism controlling for GMP (middle panel), and in FA (bottom panel) across the frontal lobe. The statistical maps used TFCE methods and are both thresholded at FWE corrected *P* < 0.05. Blue colors represent significant post-lesion decreases, whereas red/yellow colors represent significant post-lesion increases. The Y-coordinates are relative to the posterior edge of the anterior commissure.

To explore other lesion-induced metabolic changes throughout the brain, a whole brain voxel-wise analysis was performed [lesion_[post-pre]_ – control_[post-pre]_]. Because decreases in metabolism could result from tissue damage, as well as change in function, we covaried for GMP at each voxel, which should remove variance associated with pre to post alterations in tissue structure. Prior to covarying for changes in GMP probability, a significant decrease in metabolism was observed in the lesioned region. When covarying for GMP, a decrease in metabolism within the lesioned region was not observed, suggesting that gray matter loss likely accounted for the signal change in this region. Consistent with the tissue disruptions caused by aspiration lesions the GMP in the lesion region substantially decreased following the lesion (Supplementary Table S1). Interestingly, decreases in metabolism in several other prefrontal regions were observed, even when controlling for the change in GMP, suggesting that the altered signal reflects changes in NEC-related metabolism within these regions and not the result of gray matter loss per se. We observed decreased metabolism in the ventrolateral PFC (area 12/47), as well as portions of the medial and lateral OFC (areas 11 and 14) anterior to the site of the lesion (Fig. 5, middle panel, and Supplementary Table S3).

## DISCUSSION

Overall, these discrete aspiration lesions of the primate posterior OFC result in altered structure and function in components of the neural circuitry underlying AT. Previous work using this lesioning strategy called to attention the unintended structural damage resulting from the aspiration lesions, particularly to fibers passing adjacent to and through the ventral PFC [25]. Here, with anxiety phenotyping and multimodal neuroimaging, we extend earlier work demonstrating alterations in emotion processing following damage to the posterior OFC [25] to understand the consequences of discrete posterior OFC lesions on white matter tracts, gray matter integrity and metabolism in downstream targets as they relate to temperamental anxiety.

## EFFECTS OF POSTERIOR OFC LESIONS ON THREAT RESPONSES

As predicted and based on earlier work, the posterior OFC lesions resulted in reductions in anxiety-related behavior. While relatively modest in magnitude and in general, trending toward significance, it is noteworthy that reductions were observed across several different threat-related paradigms. Specifically, during the NEC paradigm, AT was reduced, with lesioned animals freezing less than controls across the entire duration of the 30-min NEC exposure. Freezing behavior was also reduced when animals were exposed to a novel conspecific, and habituation to the novel testing environment used for snake fear testing was only observed in control animals. However, the lesioned subjects did not differ from controls in their latency to retrieve a food reward in the presence of fear-inducing stimuli (real and fake snakes).

The modest behavioral effects observed are consistent with a circuit model that includes the OFC as a regulatory region that, through modulation of downstream structures, changes the probability that certain defensive repertoires will be engaged, depending on the nature of the threat [54]. Indeed, the circuit model our group has proposed for AT includes the posterior OFC as a regulatory region, which relies on connections with the extended amygdala to act as a critical intermediary between frontal systems and effector sites in the midbrain and brainstem [7].

## POSTERIOR OFC LESION INDUCED STRUCTURAL ALTERATIONS

The lesions damaged both gray and white matter within the pOFC, the extent of which was characterized *in vivo* using a variety of neuroimaging measures. First, lesions were manually traced on anatomical T1 scans (Fig. 2c) by two independent raters. While the lesions were primarily localized to gray matter that fell within posterior areas 13 and 25, the manual tracing revealed that the lesions also encompassed adjacent white matter, particularly at the medial extent of the lesion. While the tracing also showed a degree of heterogeneity among the lesions (Fig. 2c), there was not a statistically significant relationship between lesion size and the behavioral effects.

Notably, the posterior OFC lesions resulted in reductions in the microstructural integrity of the UF that were associated with the reductions in AT. The UF carries fibers that bidirectionally link posterior OFC with the anterior temporal lobe, and in our study, we found that reductions in UF FA were associated with reductions in AT. This finding is particularly relevant, as connectivity between prefrontal regions and limbic structures is one of the most commonly observed alterations across most forms of stress-related psychopathology [55, 56]. Because the UF provides structural links between the frontal and medial temporal lobes, decreased microstructural integrity of this tract could substantially affect the efficiency of communication and temporal coordination within corticolimbic networks.

Other work from our group in NHPs and humans has revealed a relation between UF FA and anxiety, such that decreased FA within the UF is associated with increased temperamental anxiety in young rhesus monkeys and anxiety disorders in adults [29]. The relationship between the integrity of the UF and anxiety was also observed in children [30] and adolescents [31]. Heritability analyses in a large sample of rhesus monkeys suggest that individual variability of this tract is largely attributable to non-heritable factors [32], pointing to the importance of environmental factors in shaping the development of this tract. Indeed, longitudinal assessments of fiber tract integrity across early development have revealed that this tract is one of the last to reach full maturity [57].

It is worth noting that the directionality of these relationships differ from those induced by the lesions in the present study: in the current study, decreased FA in the UF is associated with decreased AT, while the opposite relationship is reported in studies using neuroimaging without manipulations [30–32]. The different directionality of the findings could be related to the nature of the disruption: naturally occurring variability in UF microstructure, determined by individual differences in genetic and environmental factors, is likely qualitatively different than the complete disruption of the fibers that occurs as a result of aspiration lesions. It is also worth noting that in our previous imaging studies, the association between UF FA and anxiety was specific to males in both rhesus monkeys and children. Our study is unique in that the sample was exclusively constituted by preadolescent females, while most other lesion studies were performed in either adult cohorts, all male or of mixed sex [18, 25], or neonates [58, 59]. While the pOFC lesions only resulted in modest behavioral effects, it is possible that more robust effects would be observed as these pre-adolescent female monkeys would have matured. Related to this, it is well known that as children emerge into adolescents the risk for girls to develop anxiety disorders markedly increases compared to boys. Further work to determine the interaction between sex, development and damage to the UF will be important.

In addition to corticolimbic connections, cortico-cortical fibers connecting the ventral PFC with other PFC regions travel within the anterior portions of the UF [24, 52]. Study of patterns of connectivity within the OFC indicate a hierarchical processing structure, with posterior OFC regions preferentially receiving direct neuronal input from sensorimotor and limbic regions, whereas anterior orbitofrontal areas are primarily characterized by intra-cortical connections [13, 60]. Despite the lack of direct connections with subcortical regions implicated in affective processing, anterior OFC regions have been shown to be important in executing affective changes and associated behavioral alterations [19, 20, 61, 62], reliant upon input from subcortical structures, particularly the amygdala [63]. Thus, disruption of the posterior OFC via aspirations would not only affect the function of this subregion of the OFC but would also potentially have broad-reaching implications for more anterior portions of the OFC, as well as other connected PFC regions. Indeed, metabolism was decreased in anterior OFC and vlPFC regions while statistically controlling for the tissue altering effects of the lesions, supporting this view. In addition to metabolic changes, decreases in GMP were observed in lateral portions of the PFC. These findings are consistent with the report of Noonan *et al*. (2020) [64], who found that lesions limited to central OFC affected the activity and structure of neighboring parts of PFC. While we are hesitant to interpret the volumetric changes observed in the present study, as there is no way to disambiguate whether they result from misalignment due to lesion-related shifts of the brain or lesion-related changes in cortical volume, the overall findings nonetheless support distributed effects of these small lesions on other PFC regions.

From a treatment perspective, white matter-based plasticity is an interesting potential target for the normalization of aberrant patterns of functional connectivity within corticolimbic circuits. Studies showing experience dependent myelination of fiber tracts that structurally connect functionally interacting regions [65, 66] support the possibility that myelin-based plasticity could be a therapeutic target. By combining interventions that increase myelin-dependent plasticity with behavioral or psychotherapeutic interventions that could guide circuit-specific myelination, one could potentially leverage this experience-dependent plasticity as a treatment approach for ameliorating patterns of aberrant functional corticolimbic connectivity that characterize stress-related psychopathology. Both pharmacological [67, 68] and exercise-based interventions [69] have shown promise in increasing capacity for myelination and could be paired with psychotherapeutic and/or behavioral interventions that have shown promise in treating anxiety disorders to potentiate their therapeutic effects.

Despite these interesting findings, it is important to acknowledge the limitations of the current methods. The present results cannot disambiguate between three possible sources of behavioral and circuit-based alteration: (i) fibers of passage traveling through the pOFC region to anterior cortical targets, (ii) white matter bundles located adjacent to the cortical surface and (iii) the neurons that were directly removed as a result of the lesions. Future studies that more selectively ablate fiber tracts and/or fibers of passage are warranted. Furthermore, assessing changes in AT in subjects with excitotoxic lesions restricted to the pOFC would also be beneficial in parsing these potential mechanisms. We also emphasize that the pOFC is embedded in a complex network of brain regions, contributing to many cortical processing streams, and these lesions likely disrupted some of the connections underlying these processing streams. Selective targeting of specific projections is beginning to be possible in NHPs, using retrograde viral vectors combined with cre-recombinase systems [70]. Indeed, such studies are ongoing within our laboratory and could substantially supplement our understanding of the specific pathways disrupted by these aspiration lesions. As discussed in the following section, testing the effect of manipulating pOFC → BST fibers may be of particular interest as a novel target for modulating AT.

## EFFECTS OF POSTERIOR OFC LESIONS ON THREAT-RELATED METABOLISM

Given the reduction in structural integrity of the UF, which carries fibers from the PFC to the anterior temporal lobe, it is interesting that we did not find altered metabolism, either in the temporal cortex or in structures contained within the medial temporal lobe, such as the hippocampus and amygdala. Previous studies in our lab link metabolism within these structures to individual differences in AT [5, 6] and demonstrate a causal role for the dorsal amygdala in the expression of AT [71–73]. The posterior OFC, particularly the orbital proisocortex (OPro) and posterior portions of area 13, is densely reciprocally connected with the amygdala [60]. It is possible that activity of neurons in the amygdala was changed in a way that is not reflected in overall FDG-PET metabolism. Indeed, the encoding of features of aversive stimuli by neurons in the amygdala and OFC have different temporal dynamics [74, 75], suggesting that timing may play an important role in understanding how the amygdala encodes features related to threats. Further investigation using methods with increased spatial and temporal resolution is warranted to understand how this disruption impacted communication between frontolimbic regions.

We did, however, observe changes in metabolism in the BST, particularly its anterior portions. The BST is a structure that is often considered together with the dorsal amygdala (i.e. the central nucleus) as the extended amygdala, due to similar developmental origins, gene expression patterns and cell types [7]. The finding of decreased BST metabolism is consistent with a prior lesion study, where large aspiration lesions of the majority of the OFC resulted in decreased freezing as well as decreased metabolism in the BST [9]. The BST is intrinsically coupled with various frontal regions, including the medial PFC [76], a pattern of connectivity that is affected in individuals with anxiety disorders [77] and during shock anticipation [78]. Deterministic tractography studies and postmortem tract characterization in humans support the existence of a fiber bundle connecting the BST region with ventral prefrontal regions. Several studies suggest that projections [79, 80] from either the posterior OFC or neighboring sgACC directly innervate the BST, although a systematic characterization of these projections in primates has not been performed. In our own exploratory tractography analyses using the lesion location as an ROI, we found a fiber bundle that traveled from the ventral PFC along the midline to the BST region, consistent with direct projections between ventral PFC regions and the BST.

With respect to understanding the function of this pathway, studies in rodents have also characterized connections between the infralimbic (IL) cortex, the analog of the sgACC/posteromedial OFC, with the BST [81–83]. Interestingly, in rats, projections from the IL exert a strong excitatory influence on neurons within the BST [84], suggesting that these projections serve a modulatory role over excitation within the BST. Consistent with this, individuals with damage to the vmPFC have decreased resting blood flow to the BST [85], potentially reflecting decreased excitatory influence. Together with our results reporting decreased metabolism within the BST following posterior OFC lesions, these studies suggest that modulation of activation of the BST by various frontal regions may play an important role in threat responding. Future studies characterizing the anatomical links between the posterior OFC and BST could help to better understand the functional role of these projections in anxiety, threat responding, and the risk to develop anxiety-related psychopathology.

## CONCLUSIONS

Together, these results highlight the importance of NHP models in understanding the neural circuitry underlying temperamental anxiety, particularly with respect to the contributions of the substantially expanded PFC and the white matter tracts connecting the frontal lobe to the rest of the brain. By combining multimodal imaging with behavioral phenotyping and causal manipulations, we demonstrate that fiber disrupting lesions of the posterior OFC result in modest reductions in AT, accompanied by altered structural integrity of white matter tracts proximal to the lesion and reduced metabolism in the BST. Together, these findings provide evidence for the importance of the posterior OFC regions, as well as fibers coursing nearby and/or through the region in regulating the expression of temperamental anxiety, potentially via connections with the BST. This work supports the critical importance of the structural connectivity provided among corticolimbic structures by the UF and, combined with work demonstrating its links to temperamental anxiety and experience dependent plasticity [86], support the UF as a potential treatment target for intervention.

## Supplementary Material

supplemental video

Supplemental material

## Data Availability

The data underlying this article will be shared on reasonable request to the corresponding author.
