# Supplementary material for: Prefrontal influences on the function of the neural circuitry underlying anxious temperament in primates
Source: Oxf Open Neurosci. 2022 Oct 28;2:kvac016. doi: 10.1093/oons/kvac016 (PMC10426770; doi:10.1093/oons/kvac016)
Supplement: Web_Material_kvac016 [file web_material_kvac016.zip › pOFC_lesion_Supplement_anonymous_revised.pdf]

**Supplemental Information to accompany:**

# Prefrontal influences on the function of the neural circuitry underlying anxious temperament in primates

**Contents:**


**Figure S1:** Experimental Timeline Page 2.

**Figure S2:** Distribution of freezing behavior compared to large sample Page 3.

**Figure S3:** Latencies to reach for food reward: snake fear test. Page 4.

**Figure S4:** Latencies to reach for food reward: adaptation to reaching. Page 5.

**Table S1.** Clusters showing significant Post-lesion changes in gray matter probability. Page 6.

**Table S2.** Clusters showing significant Post-lesion changes in FA. Page 7.

**Table S3.** Clusters showing significant Post-lesion changes in brain metabolism. Page 9.

**Supplemental Movie S1**: post_lesion_movie.mp4

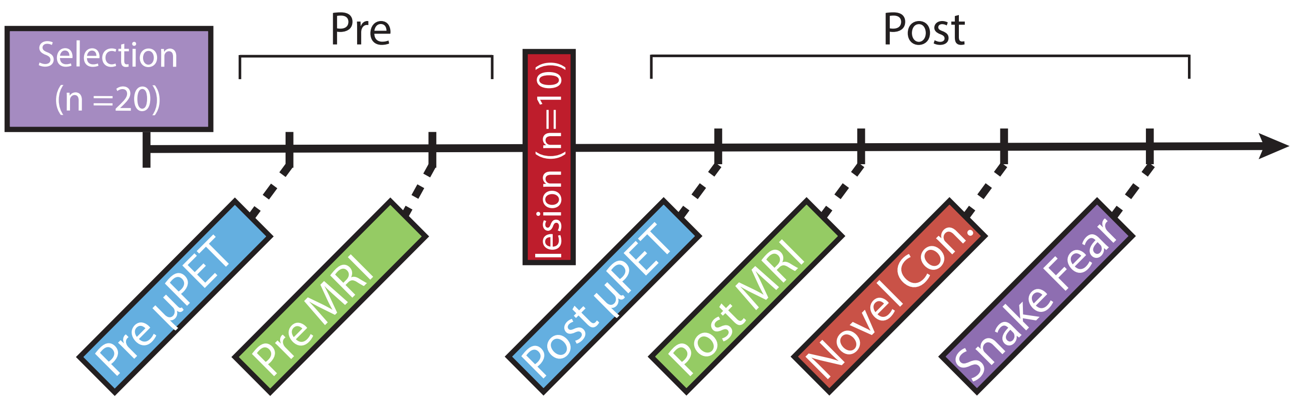


**Figure S1.** Experimental timeline. Twenty female rhesus monkeys (mean age = 2.17 (± 0.29) years) with mid- to high- levels of anxiety were selected for the experiment. All study subjects were phenotyped for their response to 30 minutes of the no-eye-contact condition (NEC) of the Human Intruder Paradigm, which was immediately followed by an FDG-PET scan to measure NEC-related brain metabolism. Subjects were then scanned with MRI (including T1-weighted and diffusion-weighted images) approximately 1 week later. These pre-surgical imaging data are referred to as 'PRE'. Half of the subjects were randomly selected to receive bilateral pOFC strip lesions, while the remaining animals served as age-matched, cage-mate controls. Following sufficient time for recovery, all animals were again assessed for behavioral and brain responses to 30 min of NEC, followed by another MRI scan approximately 1 week later. These post-surgical imaging data are referred to as 'POST'. All subjects were then tested in the “novel conspecific” paradigm, which allows for an assessment of fear and anxiety elicited during a threatening social interaction with a stimulus animal. This was followed a few weeks later by a test of the unconditioned response to a live snake. To avoid habituation to the stimulus animal and to the snake, the novel conspecific and snake fear tests, respectively, were only performed after the lesions. All procedures were performed according to the federal guidelines of animal care and use and with the approval of the University of Wisconsin-Madison Institutional Animal Care and Use Committee (IACUC).

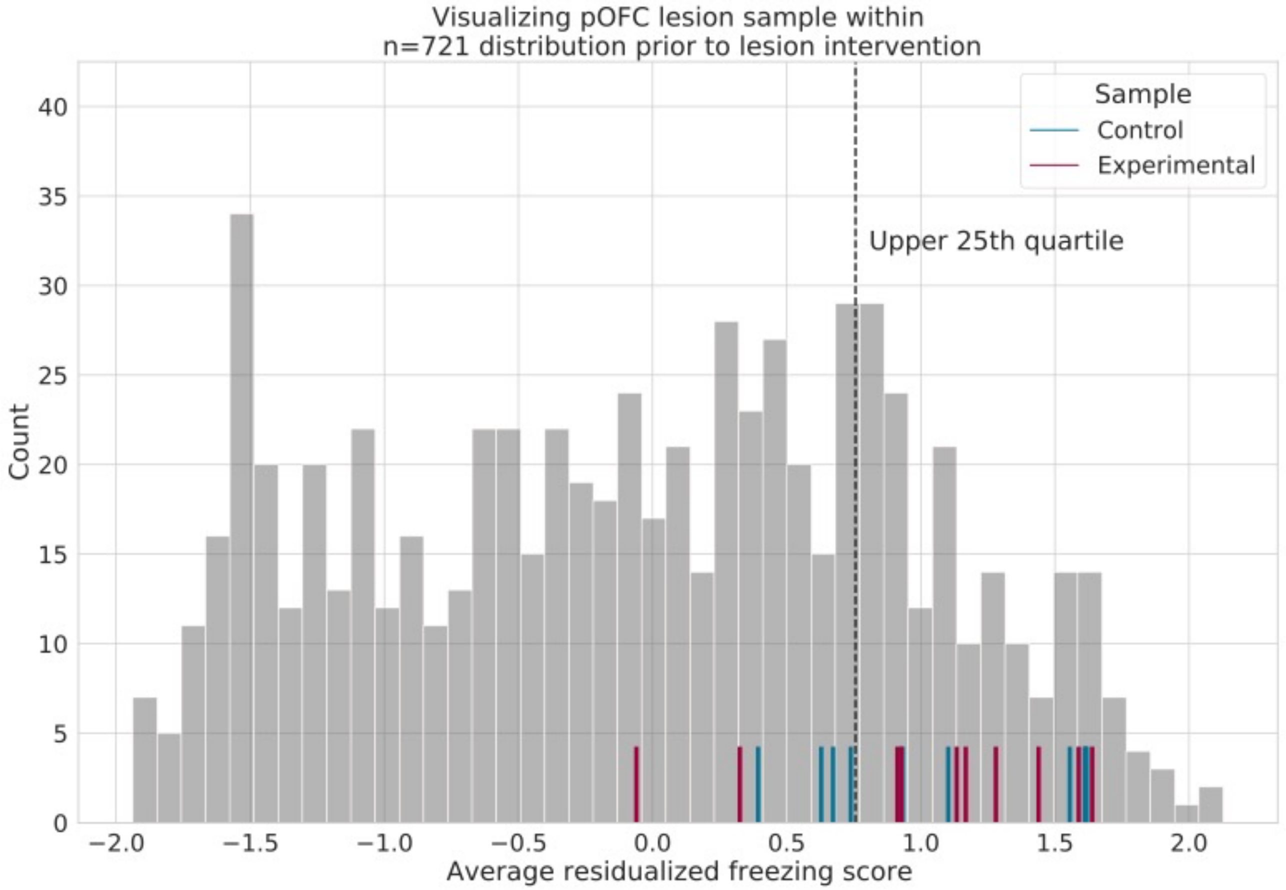


**Figure S2.** The histogram (gray bars) presents the distribution of freezing responses in a large sample (n=721) of rhesus monkeys that underwent the 30-minute NEC exposure as part of a variety of other studies performed in our laboratory. Twenty female rhesus monkeys (mean (± s.d.) age = 2.17 (± 0.29) years) with mid-to-high levels of freezing were selected for the present study. Seventy percent of the selected subjects displayed freezing responses that fell into the upper quartile of the large sample distribution.

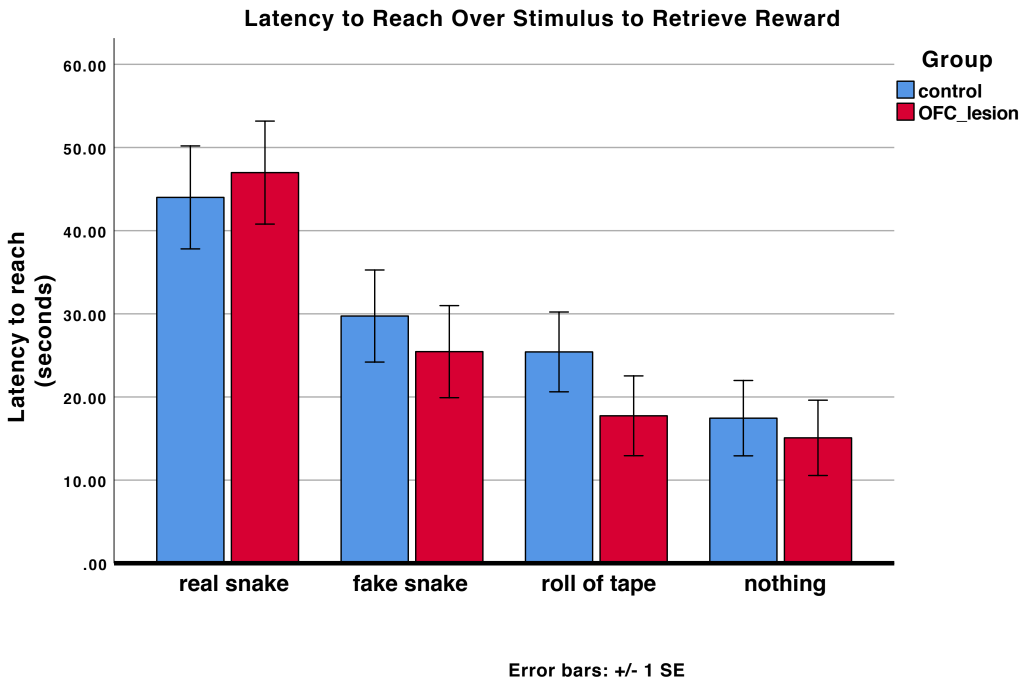


**Figure S3.** Reach Latency during snake fear testing. The monkeys displayed significantly increased latencies to reach for a food reward in the presence of a real snake (main effect of Object, $F_{(3,57)} = 27.93$, $p < 0.0001$, partial $\eta^2 = 0.59$) that did not significantly differ between groups (Object x Group interaction, $F_{(3,54)} = .941$, $p = 0.427$, partial $\eta^2=0.05$).

Reach latency during initial adaptation to testing environment

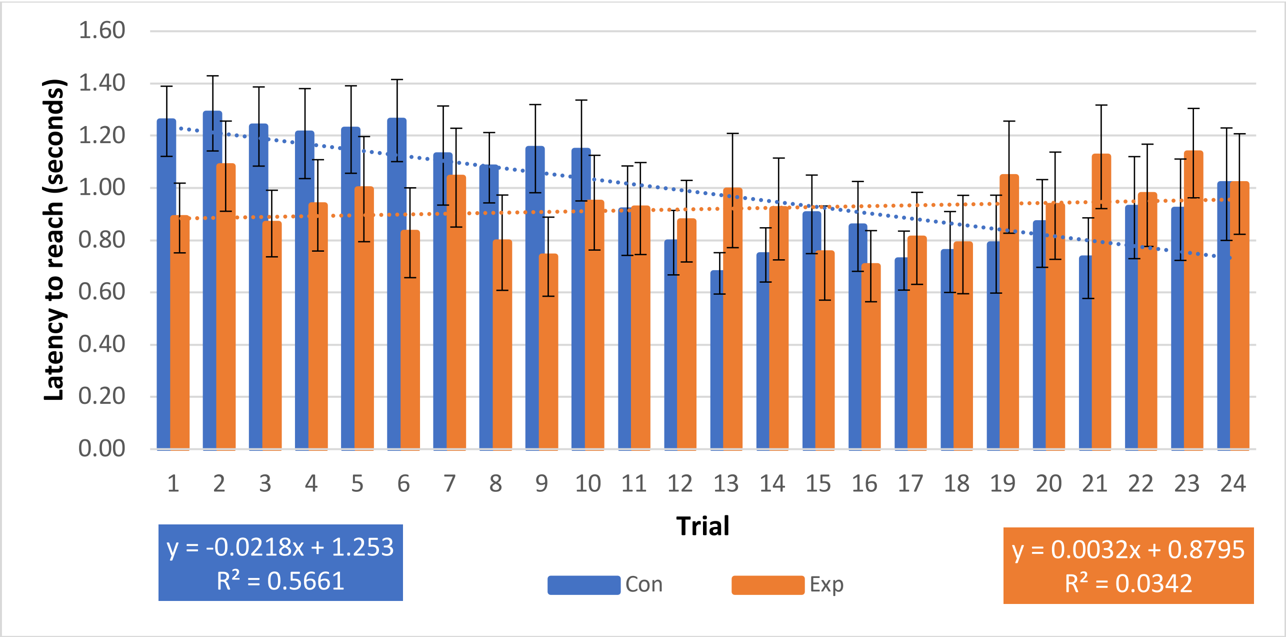


**Figure S4.** Reach latency during adaptation to snake testing environment. Subjects were trained to reach for their preferred food rewards. Differences in the time animals took to habituate to the testing environment were examined by comparing the slopes of the reach latencies across the 24 trials of the first reach adaptation training day. The control monkeys habituated rapidly to the unfamiliar conditions of the testing environment, demonstrating progressively shorter latencies to reach for a food reward, whereas the lesioned monkeys demonstrated a relatively steady response pattern (mean habituation slope, control vs. lesion, controlling for age, $F_{(1,17)} = 7.696$, $p = 0.013$, partial $\eta^2 = .31$).

**Table S1.** ***Descriptive statistics for clusters showing significant Post-lesion changes in gray matter probability.***

`

| sign | Hemisphere | Cluster | Volume (voxels) | Local Maxima | $t_{Max}$ | *tfce corrected p* | Location (mm from AC) *x* | *y* | *z* |
|---|---|---|---|---|---|---|---|---|---|
| *negative* | crosses midline | OFC, sgACC, striatum | 8305 | left Area 13a (medial orbital sulcus) | -10.35 | 0.001 | 6.25 | 14.375 | -5.00 |
| | | | | left Area 13M (middle orbital gyrus) | -9.12 | 0.001 | 9.375 | 13.125 | 0.00 |
| | | | | right Area 13a (medial orbital sulcus) | -9.52 | 0.002 | -6.25 | 14.375 | -5.00 |
| | | | | right globus pallidus | -9.73 | 0.002 | -4.375 | 1.25 | -0.625 |
| | | | | midline Area 25 | -7.95 | 0.006 | 0.00 | 10.625 | 4.375 |
| | | | | left caudate | -7.31 | 0.006 | 7.50 | -0.625 | -8.75 |
| | right | dlPFC, temporal cortex | 3369 | Area 47L (anterior part) | -10.44 | 0.001 | -11.25 | 26.25 | -6.875 |
| | | | | Area 47L (posterior part) | -6.42 | 0.008 | -20.00 | 18.75 | -6.875 |
| | | | | Area 8b | -4.63 | 0.018 | -8.125 | 15.625 | -19.375 |
| | | | | superiror temporal gyrus (ventral part) | -5.48 | 0.022 | -23.125 | 4.375 | 12.50 |
| | left | dlPFC | 1945 | Area 47L (anterior part) | -11.06 | 0.001 | 13.125 | 26.25 | -8.75 |
| | | | | Area 9/46 (principle sulcus) | -9.62 | 0.001 | 13.125 | 22.50 | -11.875 |
| | left | temporal cortex | 60 | sts (anterior part) | -7.32 | 0.032 | 21.25 | 6.25 | 15.00 |
| *positive* | crosses midline | ACC, parietal cortex, temporal cortex, occipital cortex | 75312 | left sts (posterior part), left Area V4D | 13.30 | 0.001 | 16.875 | -26.25 | -11.25 |
| | | | | left arcuate sulcus, Area 6D | 8.95 | 0.001 | 9.375 | 10.00 | -15.625 |
| | | | | right corpus callosum | 8.55 | 0.001 | -4.375 | 13.125 | -9.375 |
| | | | | left posterior insular cortex (fundus of sts) | 7.39 | 0.001 | 20.625 | -20.00 | 1.875 |
| | | | | white matter adjacent to left posterior sts | 7.46 | 0.001 | 17.50 | -16.875 | -0.625 |

Table 1. A voxelwise regression examining the ($lesion_{[post-pre]}$ – $control_{[post-pre]}$) change in gray matter probability (minimum cluster size of 50.0 $mm^3$). Abbreviations: dlPFC, dorsolateral prefrontal cortex; OFC, orbitofrontal cortex; sgACC, subgenual anterior cingulate cortex; sts, superior temporal sulcus.

**Table S2.** ***Descriptive statistics for clusters showing significant Post-lesion changes in fractional anisotropy (FA).***

| sign | Hemisphere | Cluster | Volume (voxels) | Local Maxima | $t_{Max}$ | *tfce corrected p* | Location (mm from AC) *x* | *y* | *z* |
|---|---|---|---|---|---|---|---|---|---|
| *negative* | crosses midline | dorsal striatum, bilateral anterior | 7093 | right caudate/int. capsule | -13.47 | 0.001 | -7.500 | -9.375 | 6.250 |
| | | internal capsule, external capsule, | | left caudate/int. capsule | -12.89 | 0.001 | 9.375 | -10.625 | 6.250 |
| | | white matter of the anterior cingulum, | | right ex. capsule | -5.78 | 0.001 | -11.875 | -6.250 | -1.875 |
| | | anterior corona radiata, and | | left ant. corpus callosum | -5.74 | 0.001 | 3.125 | -10.625 | 1.875 |
| | | genu of corpus callosum | | left ex. capsule | -5.68 | 0.001 | 14.375 | -6.875 | 1.250 |
| | Right | external/extreme (ex.) capsule, | 2778 | ex. capsule | -9.50 | 0.001 | -15.000 | 8.750 | 5.625 |
| | | internal capsule, thalamus, | | internal capsule | -7.18 | 0.004 | -9.375 | 5.625 | 5.625 |
| | | posterior corona radiata | | slf | -6.33 | 0.007 | -21.250 | -2.500 | 5.625 |
| | | | | VLLa | -6.20 | 0.005 | -8.750 | 7.500 | 1.250 |
| | | | | posterior corona radiata | -5.50 | 0.009 | -13.125 | 16.250 | 5.625 |
| *positive* | Left | precentral gyrus, premotor | 8776 | area 3a | 7.44 | 0.001 | 26.250 | 1.875 | 8.750 |
| | | cortex, parietal cortex | | area 4(F1) | 6.46 | 0.004 | 13.125 | 2.500 | 22.500 |
| | | superior temproral gyrus, | | TPt | 7.18 | 0.006 | 24.375 | 19.375 | 11.875 |
| | | superior temproral sulcus, | | area 3b | 5.32 | 0.007 | 27.500 | -1.875 | 4.375 |
| | | | | area 2/1 | 4.97 | 0.007 | 27.500 | -1.875 | 1.250 |
| | | | | S2E | 4.74 | 0.008 | 29.375 | 1.875 | 1.875 |
| | Left | dlPFC, vlPFC, | 4757 | inferior arcuate sulcus | 8.77 | 0.001 | 19.375 | -7.500 | 7.500 |
| | | OFC | | area 47O | 6.90 | 0.001 | 18.750 | -13.125 | -3.125 |
| | | | | superior arcuate sulcus | 5.51 | 0.006 | 12.500 | -8.125 | 16.875 |
| | | | | area 13M | 4.68 | 0.007 | 14.375 | -11.250 | -1.250 |
| | Right | postcentral gyrus, ips, cs | 2760 | PE | 7.70 | 0.008 | -10.625 | 12.500 | 21.250 |
| | | | | area 4(F1) | 5.82 | 0.019 | -3.125 | 1.250 | 23.125 |

| | | | | | | | | |
|---|---|---|---|---|---|---|---|---|
| Right | dlPFC, ps | 403 | area 9/46 | 6.66 | 0.017 | -16.875 | -6.250 | 11.250 |
| Left | ventral striatum | 386 | nucleus accumbens | 7.90 | 0.005 | 6.250 | -6.250 | -0.625 |
| | | | ventral putamen | 8.25 | 0.006 | 10.625 | -4.375 | -3.125 |
| Right | parietal cortex | 322 | PGOp | 6.02 | 0.023 | -23.750 | 18.750 | 15.000 |
| Right | ventral striatum | 166 | nucleus accumbens | 7.61 | 0.017 | -3.125 | -5.625 | 0.625 |

Table 2. A voxelwise regression examining the ($lesion_{[post\text{-}pre]}$ – $control_{[post\text{-}pre]}$) change in fractional anisotropy (minimum cluster size of 50.0 $mm^3$). Abbreviations: dlPFC, dorsolateral prefrontal cortex; AC, anterior commissure; cs, central sulcus; dlPFC, dorsolateral prefrontal cortex; ips, intraparietal sulcus; OFC, orbital prefrontal cortex; PE, parietal area PE; PGOp, opercular part of parietal area PG; S2E, external part of somatosensory area II; sfl, superior longitudinal fasciculus; TPt, temporoparietal cortex; vlPFC, ventrolateral prefrontal cortex; VLLa, ventrolateral thalamic nucleus.

**Table S3.** ***Descriptive statistics for clusters showing significant Post-lesion changes in brain metabolism.***

| sign | Hemisphere | Cluster | Volume (voxels) | Local Maxima | $t_{Max}$ | *tfce corrected p* | Location (mm from AC) *x* | *y* | *z* |
|---|---|---|---|---|---|---|---|---|---|
| *negative* | crosses midline | PFC | 4231 | midline Area 10 | -6.95 | 0.010 | 0.625 | 30.00 | -3.75 |
| | | | | left Area 46 (principal sulcus) | -7.61 | 0.011 | 10.00 | 23.75 | -9.375 |
| | | | | right Area 47O | -6.45 | 0.018 | -13.75 | 21.875 | -8.125 |
| | | | | left anterior Area 47 | -5.14 | 0.020 | 7.50 | 27.500 | -6.25 |
| | | | | left Area 14O | -5.08 | 0.021 | 0.00 | 25.625 | -0.625 |
| | | | | right Area 13 (lesion region) | -6.24 | 0.021 | -6.875 | 11.25 | 1.875 |
| | Left | striatum | 209 | medial caudate | -5.29 | 0.040 | 1.875 | 5.00 | -2.50 |
| | | | | anterior BST | -4.52 | 0.046 | 2.50 | 3.75 | -2.50 |
| | Left | vlPFC | 185 | Area 47L | -4.99 | 0.042 | 19.375 | 20.00 | -6.875 |
| *positive* | Left | central sulcus | 33 | Area 4(F1) (motor cortex) | 6.34 | 0.041 | 5.625 | -2.50 | -21.25 |

Table 3. A voxelwise regression examining the (lesion$_{[post-pre]}$ – control$_{[post-pre]}$) change in glucose metabolism while controlling for gray matter probability (minimum cluster size of 50.0 mm$^3$). Abbreviations: AC, anterior commissure; BST, bed nucleus of the stria terminalis; vlPFC, ventrolateral prefrontal cortex.
