# Supplementary material for: Prefrontal influences on the function of the neural circuitry underlying anxious temperament in primates
Source: Oxf Open Neurosci. 2022 Oct 28;2:kvac016. doi: 10.1093/oons/kvac016 (PMC10426770; doi:10.1093/oons/kvac016)
Supplement: Web_Material_kvac016 [file web_material_kvac016.zip › Supplemental file for export.docx]

**OXFNSC-2022-010.R1**

Prefrontal influences on the function of the neural circuitry underlying anxious temperament in primates

**Associate and Senior Editors feedback**

Comments to the Author:

This is an important dataset and will make an interesting paper. The editors agreed the authors need to better acknowledge the lack of effects with respect to AT when considering the lesions as a categorical manipulation. Authors also need to do more in the assessment of lesions: Figure 2 is insufficient. One silver lining is that the lack of behavioral effects might be related to variability in the lesion extent because they state in the manuscript (but do not show it anywhere) that when using the uncinate fasciculus FA reduction as a covariate, they get significant changes in AT. One suggestion is adding a paragraph discussing this and replacing the control and lesion categorical group assignments with a continuous variable related to the lesion quantification to determine if there is correlation with the anxious temperament and other behavioral measures.

**Reviewer: 1**

Comments to the Author

In this manuscript, aspiration lesions (which can also affect nearby white matter) of the posterior orbitofrontal cortex are linked to decreases in threat-related behaviors, decreases in structural integrity in the white matter of the uncinate fasciculus, and reduced metabolism in components of the circuit known to be important for threat behaviors.

This is a wonderful dataset, and I think the authors are really on the right track in terms of their hypothesized circuit, behaviors, and multimodal imaging. Ultimately, it can certainly be published. I have two broad suggestions that I think will help future readers:

First, I would like there to be much more clarity in the logic of the imaging, particularly for the white matter measurements. For example, in the abstract, the authors say that they lesions are “intended to disrupt connections between OFC and subcortical structures.” However, for the underlying pOFC, ALL connections will obviously be affected, including pOFC-cortical ones. And there are certainly some (though not all) subcortical connections from anterior/lateral OFC that will be spared—for example, many internal capsule fibers connecting the OFC to the thalamus/brainstem will already be placed too dorsally at this point to be affected by the lesion. Finally, the white matter coursing above the pOFC will include anterior/lateral OFC fibers to many cortical targets, both inside and outside the PFC. Thus, this statement is far too imprecise.

I find the paragraph in the Introduction beginning “While the effects of OFC aspiration…” to be somewhat misleading. I had thought the authors would use their data to differentiate the effects of passing fibers (those neither originating nor terminating in the posterior OFC) vs the posterior OFC itself. That does not seem to be a central component of the paper. Indeed, I wonder whether the authors predict that any of the results they found, whether behavioral or imaging, would be different with lesions that spare the nearby white matter? I suppose there is another interpretation, which has to do with the connections of the posterior OFC, in addition to the posterior OFC itself, which is more in line with the paper’s focus. In any case, I think this paragraph should be reframed as an ‘in addition to’ and should highlight all of these possibilities.

If the authors would like to make stronger claims about passing fibers vs posterior OFC fibers, they will need to perform additional analyses, such as assessing the strength of structural connectivity between other parts of PFC and the anterior temporal lobe. I don’t think this has to be done, but it might make for an interesting addition.

Along the same lines, in Figure 2, there are a few things about the visualization of the lesion extent with respect to the white matter that don’t quite make sense. In B, the sections shown pre and post lesion are not the same. More importantly, the red arrows in B don’t match the purple lesion outlines shown in C. The lesion outlines in C would seem to have only a small area of involvement of white matter (of the UF or otherwise), which we would assume would be on top of the gray matter. That is, with the exception of a very tiny bit of white matter near the midline, the purple lesion extent seems to cover no white matter. Is there more involvement of the white matter in other sections? Or should this all be interpreted in light of the very medial white matter involvement? If there are some animals that have more, perhaps additional visualizations of lesion probability would be helpful.

In Figure 2d, there is an asymmetry in the pre-lesion UF, with a hole essentially where the lesion will eventually be. Any thoughts on that? Also in Figure 2d, the red arrows on the coronal slice are pointing to fibers that are far dorsal and lateral to the purple outline in 2C.

Can more details be given on how the UF was identified? Right now, the Methods (lines 279-280) just explain, ‘using anatomically defined waypoints’ and then 2 references are given that don’t have that level of detail for monkeys. Actually, the same for all the ROI-based analyses for white matter mentioned on the following page.

My second main point relates to how this work builds upon prior work. Is the focus on white matter the main finding? If so, this should be clarified.

I think that, with fairly easy changes to the writing, this will be a very nice paper.

**Reviewer: 2**

Comments to the Author

General Comments to Authors:

This manuscript describes the behavioral, microstructural, and metabolic effects of posterior OFC aspiration lesions in preadolescent (2y) female rhesus macaques. The authors employed a battery of behavioral assessments to query features of anxious temperament (AT) including the Human Intruder paradigm, a novel conspecific test, and a behavioral reactivity test that included plastic and live snake stimuli. Brain structure and microstructure were assessed with MRI/DTI, and cerebral glucose metabolism via FDG-PET. The results suggest that the OFC lesions disrupted properties of cortico-limbic and cortico-cortico networks in these juvenile monkeys—as indicated by the significant changes in FDG and FA measures in the lesion group—but did not significantly or robustly impact AT as measured by these paradigms. Overall, this manuscript provides insights into the role of the posterior OFC in mechanisms supporting socioemotional expression of anxiety, but moreover it also highlights the broader network of structures that are involved in regulating AT during childhood/early-adolescence.

Major strengths are the comprehensive behavioral assessments and multi-modal neuroimaging. The application of FDG-PET in awake-behaving NHPs is particularly compelling in this context, as it enabled the authors to quantify patterns of brain activity during complex species-typical social behaviors that are difficult to assess with other methods (e.g. fMRI, ephys). The addition of DTI data from the same animals further corroborates the findings by illustrating a structural pathway that may be mediating the FDG effects. The major weaknesses include an over emphasis of the non-significant, trending, behavioral results; and, given the young age of the animals, a lack of acknowledgement for the developmental context of the study. These concerns are addressable.

Additional Specific Recommendations/Comments:

- As the study included only 2-year-old pre-adolescent females, the intro/discussion should be clarified to include what is known about the influence of sex/age on AT, and on OFC/UF development, in NHPs.

- In the methods, authors state that due to the small sample size and narrow age range they did not control for age in the behavioral assessment of AT (pg 8, line 185). However, for the statistical analysis of the imaging data associated with the same animals, age was included. Please explain the reason for the two different approaches and/or adjust to make them parallel.

- In the results, authors report faster habituation (progressively shorter retrieval latencies) in the OFC group compared to controls (pg 16 line 402-4). Supplementary Figure 4 illustrates these results, but the OFC group (orange bars) appears to have a steady response pattern (regression line is flat, slope = 0.0032), whereas the Controls (blue bars) appear to get faster over time (regression line has negative slope = -0.0218). Please clarify. Additionally, please add x- and y-axis labels to figure S4.

- By how much is FA lowered? Please provide some quantification of the magnitude of the significant FA changes in each group. Additionally, are the FA changes associated with reductions in AD? increases in RD? etc… And what do the DTI results suggest about the microstructural white matter changes that were induced by the lesion (myelin loss, axonal degradation, etc)? Lastly, please provide a short discussion on the interpretation of areas with significant FA increases.

- For the FDG methods, please specify if the animals were fasted prior to FDG administration. Additionally, were there any group differences in the amount of activity administered that could be confounding the results? Please provide a supplementary table illustrating mCi doses for each animal, and group statistical comparisons of mCi doses.

- For the FDG results, authors report areas of reduced glucose metabolism anterior to the lesion in areas 14 and 11 of the OFC (pg 19 line 486), but this is not reflected in Figure 4: the coronal slice illustrated that is anterior to the level of the lesion (first column, second row) does not appear to have any areas of significant metabolic reduction in the OFC. Please adjust the figure to illustrate these results more clearly.

**Decision letter first revision**

From: aizquie@psych.ucla.edu

To:oler@wisc.edu

Subject: Oxford Open Neuroscience - Decision on Manuscript ID OXFNSC-2022-010

14-Aug-2022

Dear Dr. Oler,

Manuscript ID OXFNSC-2022-010 entitled "Prefrontal influences on the function of the neural circuitry underlying anxious temperament in primates" which you submitted to the Oxford Open Neuroscience, has been reviewed. The comments of the reviewer(s) are included at the bottom of this letter.

Please note that this journal operates with transparent peer review. This means that if your submission is accepted for publication, the full peer review history of your article will publish online alongside your article. This includes reviewer comments, editor decision letters, and your author responses.

The reviewer(s) have recommended publication, but also suggest some revisions to your manuscript. Therefore, I invite you to respond to the reviewer(s)' comments and revise your manuscript.

To revise your manuscript, log into https://mc.manuscriptcentral.com/oxfnsc and enter your Author Centre, where you will find your manuscript title listed under "Manuscripts with Decisions." Under "Actions," click on "Create a Revision." Your manuscript number has been appended to denote a revision.

You may also click the below link to start the revision process (or continue the process if you have already started your revision) for your manuscript. If you use the below link you will not be required to login to ScholarOne Manuscripts.

*** PLEASE NOTE: This is a two-step process. After clicking on the link, you will be directed to a webpage to confirm. ***

https://mc.manuscriptcentral.com/oxfnsc?URL_MASK=818468d6a0ee4c59807ff2e265a9b12e

Please highlight the changes to your manuscript within the document by using the track changes mode in MS Word or by using bold or colored text.

Once the revised manuscript is prepared, you can upload it and submit it through your Author Centre.

When submitting your revised manuscript, you will be able to respond to the comments made by the reviewer(s) in the space provided. You can use this space to document any changes you make to the original manuscript. In order to expedite the processing of the revised manuscript, please be as specific as possible in your response to the reviewer(s).

IMPORTANT: Your original files are available to you when you upload your revised manuscript. Please delete any redundant files before completing the submission.

Because we are trying to facilitate timely publication of manuscripts submitted to the Oxford Open Neuroscience, your revised manuscript should be uploaded as soon as possible. If it is not possible for you to submit your revision in a reasonable amount of time, we may have to consider your paper as a new submission.

Once again, thank you for submitting your manuscript to the Oxford Open Neuroscience and I look forward to receiving your revision.

Sincerely,

Dr. Alicia Izquierdo

Senior Editor, Oxford Open Neuroscience

aizquie@psych.ucla.edu

Associate and Senior Editors feedback

Comments to the Author:

This is an important dataset and will make an interesting paper. The editors agreed the authors need to better acknowledge the lack of effects with respect to AT when considering the lesions as a categorical manipulation. Authors also need to do more in the assessment of lesions: Figure 2 is insufficient. One silver lining is that the lack of behavioral effects might be related to variability in the lesion extent because they state in the manuscript (but do not show it anywhere) that when using the uncinate fasciculus FA reduction as a covariate, they get significant changes in AT. One suggestion is adding a paragraph discussing this and replacing the control and lesion categorical group assignments with a continuous variable related to the lesion quantification to determine if there is correlation with the anxious temperament and other behavioral measures.

Reviewer: 1

Comments to the Author

In this manuscript, aspiration lesions (which can also affect nearby white matter) of the posterior orbitofrontal cortex are linked to decreases in threat-related behaviors, decreases in structural integrity in the white matter of the uncinate fasciculus, and reduced metabolism in components of the circuit known to be important for threat behaviors.

This is a wonderful dataset, and I think the authors are really on the right track in terms of their hypothesized circuit, behaviors, and multimodal imaging. Ultimately, it can certainly be published. I have two broad suggestions that I think will help future readers:

First, I would like there to be much more clarity in the logic of the imaging, particularly for the white matter measurements. For example, in the abstract, the authors say that they lesions are “intended to disrupt connections between OFC and subcortical structures.” However, for the underlying pOFC, ALL connections will obviously be affected, including pOFC-cortical ones. And there are certainly some (though not all) subcortical connections from anterior/lateral OFC that will be spared—for example, many internal capsule fibers connecting the OFC to the thalamus/brainstem will already be placed too dorsally at this point to be affected by the lesion. Finally, the white matter coursing above the pOFC will include anterior/lateral OFC fibers to many cortical targets, both inside and outside the PFC. Thus, this statement is far too imprecise.

I find the paragraph in the Introduction beginning “While the effects of OFC aspiration…” to be somewhat misleading. I had thought the authors would use their data to differentiate the effects of passing fibers (those neither originating nor terminating in the posterior OFC) vs the posterior OFC itself. That does not seem to be a central component of the paper. Indeed, I wonder whether the authors predict that any of the results they found, whether behavioral or imaging, would be different with lesions that spare the nearby white matter? I suppose there is another interpretation, which has to do with the connections of the posterior OFC, in addition to the posterior OFC itself, which is more in line with the paper’s focus. In any case, I think this paragraph should be reframed as an ‘in addition to’ and should highlight all of these possibilities.

If the authors would like to make stronger claims about passing fibers vs posterior OFC fibers, they will need to perform additional analyses, such as assessing the strength of structural connectivity between other parts of PFC and the anterior temporal lobe. I don’t think this has to be done, but it might make for an interesting addition.

Along the same lines, in Figure 2, there are a few things about the visualization of the lesion extent with respect to the white matter that don’t quite make sense. In B, the sections shown pre and post lesion are not the same. More importantly, the red arrows in B don’t match the purple lesion outlines shown in C. The lesion outlines in C would seem to have only a small area of involvement of white matter (of the UF or otherwise), which we would assume would be on top of the gray matter. That is, with the exception of a very tiny bit of white matter near the midline, the purple lesion extent seems to cover no white matter. Is there more involvement of the white matter in other sections? Or should this all be interpreted in light of the very medial white matter involvement? If there are some animals that have more, perhaps additional visualizations of lesion probability would be helpful.

In Figure 2d, there is an asymmetry in the pre-lesion UF, with a hole essentially where the lesion will eventually be. Any thoughts on that? Also in Figure 2d, the red arrows on the coronal slice are pointing to fibers that are far dorsal and lateral to the purple outline in 2C.

Can more details be given on how the UF was identified? Right now, the Methods (lines 279-280) just explain, ‘using anatomically defined waypoints’ and then 2 references are given that don’t have that level of detail for monkeys. Actually, the same for all the ROI-based analyses for white matter mentioned on the following page.

My second main point relates to how this work builds upon prior work. Is the focus on white matter the main finding? If so, this should be clarified.

I think that, with fairly easy changes to the writing, this will be a very nice paper.

Reviewer: 2

Comments to the Author

General Comments to Authors:

This manuscript describes the behavioral, microstructural, and metabolic effects of posterior OFC aspiration lesions in preadolescent (2y) female rhesus macaques. The authors employed a battery of behavioral assessments to query features of anxious temperament (AT) including the Human Intruder paradigm, a novel conspecific test, and a behavioral reactivity test that included plastic and live snake stimuli. Brain structure and microstructure were assessed with MRI/DTI, and cerebral glucose metabolism via FDG-PET. The results suggest that the OFC lesions disrupted properties of cortico-limbic and cortico-cortico networks in these juvenile monkeys—as indicated by the significant changes in FDG and FA measures in the lesion group—but did not significantly or robustly impact AT as measured by these paradigms. Overall, this manuscript provides insights into the role of the posterior OFC in mechanisms supporting socioemotional expression of anxiety, but moreover it also highlights the broader network of structures that are involved in regulating AT during childhood/early-adolescence.

Major strengths are the comprehensive behavioral assessments and multi-modal neuroimaging. The application of FDG-PET in awake-behaving NHPs is particularly compelling in this context, as it enabled the authors to quantify patterns of brain activity during complex species-typical social behaviors that are difficult to assess with other methods (e.g. fMRI, ephys). The addition of DTI data from the same animals further corroborates the findings by illustrating a structural pathway that may be mediating the FDG effects. The major weaknesses include an over emphasis of the non-significant, trending, behavioral results; and, given the young age of the animals, a lack of acknowledgement for the developmental context of the study. These concerns are addressable.

Additional Specific Recommendations/Comments:

- As the study included only 2-year-old pre-adolescent females, the intro/discussion should be clarified to include what is known about the influence of sex/age on AT, and on OFC/UF development, in NHPs.

- In the methods, authors state that due to the small sample size and narrow age range they did not control for age in the behavioral assessment of AT (pg 8, line 185). However, for the statistical analysis of the imaging data associated with the same animals, age was included. Please explain the reason for the two different approaches and/or adjust to make them parallel.

- In the results, authors report faster habituation (progressively shorter retrieval latencies) in the OFC group compared to controls (pg 16 line 402-4). Supplementary Figure 4 illustrates these results, but the OFC group (orange bars) appears to have a steady response pattern (regression line is flat, slope = 0.0032), whereas the Controls (blue bars) appear to get faster over time (regression line has negative slope = -0.0218). Please clarify. Additionally, please add x- and y-axis labels to figure S4.

- By how much is FA lowered? Please provide some quantification of the magnitude of the significant FA changes in each group. Additionally, are the FA changes associated with reductions in AD? increases in RD? etc… And what do the DTI results suggest about the microstructural white matter changes that were induced by the lesion (myelin loss, axonal degradation, etc)? Lastly, please provide a short discussion on the interpretation of areas with significant FA increases.

- For the FDG methods, please specify if the animals were fasted prior to FDG administration. Additionally, were there any group differences in the amount of activity administered that could be confounding the results? Please provide a supplementary table illustrating mCi doses for each animal, and group statistical comparisons of mCi doses.

- For the FDG results, authors report areas of reduced glucose metabolism anterior to the lesion in areas 14 and 11 of the OFC (pg 19 line 486), but this is not reflected in Figure 4: the coronal slice illustrated that is anterior to the level of the lesion (first column, second row) does not appear to have any areas of significant metabolic reduction in the OFC. Please adjust the figure to illustrate these results more clearly.

Date Sent: 14-Aug-2022

**Author's Response to Decision Letter for (OXFNSC-2022-010)**

Below please find our responses to the reviews in the Decision Letter. We have also uploaded this response as a 'Supplemental File for Review', which contains images and formatting not provided in the text below.

---

We thank the Editors for the feedback. Per the Reviewers’ suggestions, we looked to see if, in the lesion group, there was an association between lesion volume (assessed via manual tracing) and changes in AT/other behavioral measures. The relationship between the change in AT and lesion volume was not significant (R2 = 0.009, p = 0.79). Non-significant associations were also found with lesion volume and the changes in cortisol (R2 = 0.07, p = 0.45) and in freezing (R2 = 0.08, p = 0.43). We note that there are only 10 subjects in the lesion group, making it difficult to interpret null results. We also note that the volume may not be the best reflection of lesion extent - these lesions were performed in a region with several distinct cytoarchitectonic areas (14, posterior 13, some 25). The lesions also captured slightly different amounts of white matter adjacent to the cortical surface across subjects. Indeed, the observation that the reduction in AT was associated with the change in uncinate FA would be in line with an interpretation that the white matter damage is more central to the behavioral effects observed. Although it is reasonable to assume that larger lesions would cover more of these distinct features of the pOFC region, the lack of an association between lesion extent and behavioral measures suggests that maintaining the two groups as separate is a more statistically robust approach, especially when considering the sample size. We have added the results of these new correlational analyses to the Results section on page 17 and to the Discussion on page 21.

Replies to Reviewer #1

Reviewer 1, Point 1: We thank the reviewer for their points regarding fibers traveling in the uncinate fascicle. We agree that the uncinate fascicle connects frontal cortex with several parts of the inferior temporal cortex, which we neglected to mention. We have now altered the text in the abstract to correct the misleading statement. See page 1.

Reviewer 1, Point 2: We thank the reviewer for this feedback and have amended the text to reflect this more nuanced interpretation (see page 2). In addition, we have added a section to the Discussion that considers these interpretation issues. As for the logic of the study, it is true that if the pOFC strip lesions had a significant effect on AT and other threat-related responses, as we had anticipated, we could conclude only that either posterior OFC, the fibers traveling near or through posterior OFC, or the two together were responsible for the effect on behavior. Future studies would need to test whether posterior OFC itself contributes, perhaps by examining the effects on AT of excitotoxic lesions of posterior OFC or the use of reversable inactivation with chemogenetic methods.

Reviewer 1, Point 3: We appreciate the reviewer’s comment. Rather than pursue the structural connectivity between PFC and temporal lobe, we have taken the Editor’s suggestion regarding the possible relationship between the extent of the lesion and AT. Please see the response to editor for a discussion of those findings.

Reviewer 1, Point 4: We thank the referee for his/her constructive comments regarding Figure 2. For B, we chose sections for pre- and post-lesion that were as closely matched as possible. The sections cannot be ‘identical’ because they are not from the same scan session, and scan images may not be in exactly the same plane. (The scans were acquired ~4.5 months apart.)

The red arrows in B were intended to point the reader to the general location of the lesion. We have removed the arrows so the readers can determine for themselves the differences between the images.

In C, the purple lines demonstrated the best estimate of gray matter loss due to the lesion. Based on Nissl-stained material, it is difficult to identify white matter loss, except medially where the lesion intentionally includes white matter (as well as gray matter) in the most ventromedial portion of the frontal lobe, next to the olfactory tract. That is why the tractography and imaging in this study is essential to assessing the lesion. After reviewing the material again, we agree with the referee that the purple lines in C did not accurately reflect the extent of the lesion. The banks of the medial orbital sulcus should have been included. We have now corrected this in C in a revised Figure 2, which includes several additional visualizations of the lesions at different coronal planes. In an effort to better demonstrate individual variability in lesion extent, we have also amended the figure to show the overlap between the 10 subjects.

The reviewer is correct in pointing out that much of the white matter directly covered by the lesion is at the very medial portion of the lesion, which was damaged in a majority of subjects. While small, elegant tracing studies done by Dr. Suzanne Haber and colleagues have shown that this portion of the ventral prefrontal white matter contains many fibers exiting the ventral PFC, as well as fibers traveling from anterior portions of the ventral PFC to other white matter bundles. Thus, it is possible that disruption of this small region did in fact make a substantial contribution to the behavioral and metabolic changes observed following the lesions. Of course, it is not possible to directly support that claim with the current data, but further studies that are more targeted to this portion of the ventral prefrontal white matter may prove informative.

Reviewer 1, Point 5: Previous studies have shown an asymmetry in the size of the UF in humans (Highley et al., 2002, Hau et al., 2017), with the right side larger than the left, which also appears to be the case in our data set. However, the metrics that we use for testing represent an averaged FA value across the entire extent of the tract, which should remove any influence of differential size across the hemispheres. At the Pre time point, there is not a statistically significant difference between average FA across the two hemispheres (t = -1.62, p = 0.12). The same is true after the lesion, both within the entire sample (t = -0.63, p = 0.54) and in the lesion group only (t = -1.18, p = 0.27). Finally, there is not a statistically significant difference in lesion volume across hemispheres (t = -1.31, p = 0.22), justifying our decision to average across the hemispheres for our analyses.

In addition to the changes to the lesion visualization discussed above, we have removed the arrows so the readers can determine for themselves the differences between the images. We are unsure of the exact cause of what appears to be a hole in the anterior portion of the average UF at the pre-lesion timepoint. One possibility is that this portion of the UF, which has entered the ventromedial PFC, here crosses with other cortico-cortical fiber bundles that are having an impact on the deterministic tractography reconstruction in this sample. We are reassured, however, that the ‘hole’ is visible at both the pre- and post-lesion time points, and that it is considerably larger after the lesion. We now included a supplemental video that shows the 3D rendering of the UF with respect to the lesion. We hope that this video helps in understanding the extent and placement of the lesion relative to the UF fibers.

Reviewer 1, Point 6: We have updated the methods to include a more detailed description of the methods for defining the UF, as well as included relevant references (see page 12). We added the following section: “More specifically, the most posterior coronal section that showed clear separation of the frontal and temporal lobes bilaterally was identified in the population average Pre-surgery FA image. Bilateral frontal and temporal lobe seed regions of interest were then manually drawn on this coronal section (see figure in supplemental file). The Boolean AND term was used to select only fibers that crossed through both the temporal and frontal seed regions of interest.”

Reviewer 1, Point 7: This work builds on prior work by testing whether white matter traveling in/near posterior OFC contributes to AT. As explained above, if the posterior OFC strip lesions had a significant effect on AT and other threat-related responses, as we had anticipated, we could conclude only that either posterior OFC, the fibers traveling near or through posterior OFC, or the two together were responsible for the effect on behavior. Future studies would need to test whether posterior OFC itself contributes, perhaps by examining the effects on AT of discreet excitotoxic lesions of OFC. We have included a consideration of these points and limitations of interpretation in the Discussion on page 24.

Replies to Reviewer #2

Reviewer 2, Point 1: In response to the referee’s comments, we have expanded our description of what is known about sex and age influences on AT in macaque monkeys in the Introduction on page 3, and in the Discussion on page 23.

Reviewer 2, Point 2: We apologize for this oversight. We have updated the statistics to include age in the behavioral analyses. These changes can be found throughout the manuscript.

Reviewer 2, Point 3: We agree that the interpretation of the reach latency data was potentially misleading and thank the reviewer for catching this oversight. We have changed the text in the Results section on page 16 to describe the observed effect more parsimoniously. The figure caption in the Supplement has been changed as well. We have also added axis labels to the Supplemental figure.

Reviewer 2, Point 4: In order to address the question regarding the magnitude of the FA decrease, we have amended Figure 3 to include a bar graph of the average FA change across the entire UF.

As can be seen in the heatmap in supplemental file, in the 10 lesioned animals, the change in FA (averaged across hemispheres) was positively associated with the change in AD (r = .093, p = 0.79), and inversely associated with changes in RD and TR, and though not significant, are in the expected direction. We believe the pattern of changes in these metrics is most likely indicative of axonal degeneration, consistent with prior findings of axonal degeneration following aspiration lesions (Butters et al., 1973). Interestingly, these prior studies also observed degeneration extending in the MD thalamus, consistent with our findings of both a volumetric reduction and decrease in FDG metabolism in this region. Unfortunately, the prior studies did not assess fiber degeneration in the region of the BST. In the future, we plan to characterize the morphological properties of this region ex vivo to compare with in vivo results.

With respect to the increase in FA, it is possible that these changes reflect compensatory mechanisms within white matter pathways, or gray matter degradation in the cortex. Many of these increases are restricted to grey matter, particularly in dorsolateral areas, perhaps reflecting decreased engagement of these regions following disruption to the ventral and medial cortical networks. However, we should note that we did not observe changes in FDG metabolism in these frontal regions during exposure to the NEC. If anything, decreases in FA in more anterior PFC regions displayed changes in metabolism (decreases) that were in the same direction as anterior OFC regions. Since we did not perform any tasks with these subjects that directly tested engagement of the dlPFC, such as spatial working memory tasks, we are hesitant to make concrete claims about changes in functional recruitment of these areas. We have added a brief discussion of these results on page 19 and thank the reviewer for raising this issue.

Reviewer 2, Point 5: The animals were food fasted prior to FDG administration; this detail has been added to Methods on page 9. As to the question regarding potential group differences in the amount of radioactivity administered, we ran a repeated measures ANOVA to examine this possibility, and as expected there was no main effect of Time (Pre vs. Post), no main effect of Group and no interaction with regard to the FDG doses administered during the experiment (see graph of results the requested table in supplemental file).

Reviewer 2, Point 6: We thank the reviewer for their feedback on this point and have adjusted Figure 4 (now Figure 5) to include a coronal slice that more accurately reflects the decrease in metabolism in portions of the anterior OFC, both medial (area 11) and lateral (area 47). We hope this satisfies the reviewers' concern.

Citations:

Butters, N., Butter, C., Rosen, J. & Stein, D. Behavioral effects of sequential and one-stage ablations of orbital prefrontal cortex in the monkey. Exp. Neurol. 39, 204–214 (1973).

Hau J, Sarubbo S, Houde JC et al. Revisiting the human uncinate fasciculus, its subcomponents and asymmetries with stem-based tractography and microdissection validation. Brain Struct Funct 2017;222:1645–62.

Highley JR, Walker MA, Esiri MM et al. Asymmetry of the Uncinate Fasciculus: A Post-mortem Study of Normal Subjects and Patients with Schizophrenia. Cerebral Cortex 2002;12:1218–24.

**Decision Letter (OXFNSC-2022-010.R1)**

From: aizquie@psych.ucla.edu

To: oler@wisc.edu

CC: aizquie@psych.ucla.edu

Subject: Oxford Open Neuroscience - Decision on Manuscript ID OXFNSC-2022-010.R1

18-Oct-2022

Dear Dr. Oler,

It is a pleasure to accept your revised manuscript entitled "Prefrontal influences on the function of the neural circuitry underlying anxious temperament in primates" in its current form for publication in Oxford Open Neuroscience. Comments are included at the foot of this letter.

Please note that this journal operates with transparent peer review. This means that the full peer review history of your article will publish online alongside your article. This includes reviewer comments, editor decision letters, and your author responses.

Thank you for your contribution! On behalf of the Editors of Oxford Open Neuroscience, we look forward to your continued support of the Journal.

Sincerely,

Dr. Alicia Izquierdo

Senior Editor, Oxford Open Neuroscience

aizquie@psych.ucla.edu

Associate Editor

Comments to the Author:

I would like to thank the authors for thoughtfully responding to the comments made by the editors and reviewers. The overall effects of the posterior OFC “strip” lesions and such a full characterization of the functional and structural changes is rare and important. I don’t see a need to send the manuscript out for further review and am happy to accept it for publication.

Date Sent:

18-Oct-2022
